# Seasonal and annual variability of coastal sulphur plumes in the northern Benguela upwelling system

**DOI:** 10.1371/journal.pone.0192140

**Published:** 2018-02-08

**Authors:** Thomas Ohde, Isabelle Dadou

**Affiliations:** Laboratoire d’Etudes en Géophysique et Océanographie Spatiales (LEGOS), University of Toulouse, CNES, CNRS, IRD, UPS, Toulouse, France; University of Vigo, SPAIN

## Abstract

We investigated the seasonal and annual variability of surface sulphur plumes in the northern Benguela upwelling system off Namibia because of their significant impacts on the marine ecosystem, fishing industry, aquaculture farming and tourism due to their toxic properties. We identified the sulphur plumes in ocean colour satellite data of the medium resolution imaging spectrometer (MERIS) for the 2002–2012 time period using the differences in the spectral properties of Namibian Benguela optical water types. The sulphur events have a strong seasonal cycle with pronounced main and off-seasons forced by local and remote-driven processes. The main peak season is in late austral summer and early austral autumn at the beginning of the annual upwelling cycle caused by increasing equatorwards alongshore winds. The sulphur plume activity is high between February and April during the seasonal oxygen minimum associated with the seasonal reduction of cross-shore ventilation of the bottom waters, the seasonal southernmost position of the Angola Benguela Frontal Zone, the seasonal maximum of water mass fractions of South Atlantic and Angola Gyre Central Waters as well as the seasonal arrival of the downwelling coastal trapped waves. The off-season is in austral spring and early austral summer during increased upwelling intensity and enhanced oxygen supply. The annual variability of sulphur events is characterized by very high activities in years 2004, 2005 and 2010 interrupted by periods of lower activity in years 2002 to 2003, 2006 to 2009 and 2011 to 2012. This result can be explained by the relative contributions or adding effects of local and remote-driven forces (from the equatorial area). The probability for the occurrence of sulphur plumes is enhanced in years with a lower annual mean of upwelling intensity, decreased oxygen supply associated with decreased lateral ventilation of bottom waters, more southern position of the Angola Benguela Frontal Zone, increased mass fraction of South Atlantic Central Water and stronger downwelling coastal trapped waves. Understanding of the variability and forcing processes of the toxic sulphur events will help in the future to monitor and forecast them as well as to manage their social and economic consequences in the northern Benguela upwelling system off Namibia.

## Introduction

The Benguela upwelling system (BUS) is one of the four eastern boundary upwelling systems of the global ocean. The BUS sustains a very high primary production associated with the upwelling of nutriment rich waters forced by the trade winds [1–3]. Due to the subsequent export production and the intense remineralisation, an oxygen minimum zone (OMZ) is present in the northern Benguela upwelling system (NBUS) where the trade winds are permanent all the year [4–6]. In the NBUS off Namibia associated with the OMZ, hydrogen sulphide outbreaks and their sulphur plumes are unique events not found anywhere else in the global ocean with such intensity. These events, which influence the marine ecosystem due to their toxic effects [7, 8], have direct impacts on the biogeochemical cycles [9, 10] and are able to affect the Namibian industry [11–13]. When hydrogen sulphide (H_2_S) is produced and consumed in the OMZ, several biogeochemical reactions take place and have immediate influences on the oxygen, sulphur and nitrogen cycles [6, 9, 10]. The local fish and shellfish industry, an important Namibian economic factor, is threatened. These events can decrease the amount of benthos, fish stocks and other marine organisms and can cause mass mortalities of commercially important fishes, oysters, crabs, shrimps and prawns [11, 14, 15]. Moreover, the local population is injured to the unpleasant smell and corrosive effects [16]. The tourism activity is also impacted due to the accumulations of dead marine organisms on the beaches [11, 14].

The spatial-temporal variability of these events and their forcing are poorly understood. However, the formation of H_2_S is mainly attributed to the anaerobic decomposition of organic matter by bacterial sulphate reduction [17, 18] in the diatomaceous mud belt of the central shallow Namibian shelf maintained by the very high productivity [19–21]. The bacterial sulphate reduction in the water column contributes little to the accumulation of H_2_S in the water column [9]. The initiation of sulphidic water is assigned to either the eruptive-spontaneous flux of methane carrying H_2_S from the gas-loaded shelf sediments [16, 22, 23] or the diffusive-uniform flux of H_2_S from sulphidic sediments [9, 24]. The flux from the sediment to the bottom water layer is controlled by sulphide-oxidising bacteria [25]. The process of H_2_S-consumption by the large sulfur and nitrate-storing sulphur bacteria Thiomargarita namibiensis, Thioploca, and Beggiatoa is well-known but their regulation mechanisms are poorly understood. However, for the development of bottom water sulphidic conditions, this bacterial sulphide oxidation must be ineffective mainly after complete depletion of nitrate in the bottom water [9]. The oxygen level on the Namibian shelf plays also a key role. Thiomargarita namibiensis can live under low oxygen or anoxic conditions [25]. However, atmospheric oxygen levels are toxic for Beggiatoa [26] and Thioploca [27]. The anaerobic process of organic matter decomposition occurs mainly below the sediment-water interface during anoxic conditions supporting the formation of H_2_S [17, 28, 29]. The oxygen balance over the Namibian shelf depends on the water mass composition and the biological activities. The water mass composition is controlled by large scale and local circulation processes. The main large scale driver of oxygen variability on the Namibian shelf is the strength and duration of the poleward undercurrent [30]. The local acting process of cross-shelf circulation forced mainly by trade winds ventilates also the shelf water [9]. Biological activities control also the oxygen level through the consumption of oxygen during the oxic remineralisation of the organic matter (e.g., [4]).

Investigations have shown that sulphidic water can be detoxified by chemolithotrophic bacteria to harmless colloidal sulphur (S^0^) [10]. The frequency, variability and intensity of the occurrence of this detoxification process are not well-known. However, the complete and incomplete chemolithotrophic oxidation of sulphide occurs in specific subsurface water layers under nitrate-enriched and nitrate-limiting conditions, respectively [10]. Nevertheless, the sources of the sulphur plumes in the surface water layer can be the H_2_S enriched bottom water coming from the different fluxes of H_2_S from the sulphidic sediments [9, 10, 22–24]. Another source could be the S^0^ enriched subsurface water coming from the detoxification process [10]. The H_2_S enriched bottom water can be upwelled at the coast during the onset of upwelling favorable wind forces [31]. Observations of crater structures at the seafloor [9, 22] and remote-sensed offshore sulphur patches [16, 23] suggest that massive eruptions of H_2_S can also be a potential source. These individual and rare eruptions may have a strong impact on local scales but with less regional significance [9]. The short-term presence of H_2_S in the upper water and lower atmosphere layers is evident because local inhabitants reported the intensive smell and the corrosive effects of H_2_S [16]. Measurements in the uppermost water layers demonstrated that the dissolved H_2_S can be oxidised to S^0^ by biological reactions (Namibian shelf: [10]; Peru-Chile shelf: [32, 33]; Chesapeake Bay: [34, 35]) and chemical reactions (Namibian shelf: [16, 23]). Some of the sulphidic events can be overlooked by remote sensing if the sulphide is completely consumed by chemolithotrophic bacteria in the subsurface waters before it is upwelled to the water surface or if the S^0^ remains in the subsurface and deep water [10].

The main focus of this paper is the investigation of the coastal sulphur plumes in the surface water layer in the NBUS off Namibia. At the moment, there are large gaps in the knowledge about these sporadic events. Up to now their temporal variability as well as their forcing by local and remote-driven processes are not well-known. Few studies exist describing their frequency, intensity and size, and moreover, they are limited to short time periods. The objective of this paper is to investigate the seasonal and annual variability of coastal sulphur plumes in relation to their forcing using remote sensing data sets combined with in-situ measurements. The focus is on the full lifetime period of the medium resolution imaging spectrometer (MERIS) on board the ENVISAT satellite of the European Space Agency (ESA) from 2002 to 2012 (10 years). In particular, the aim is to quantify their spatial extension and intensity on the basis of water-leaving reflectance of MERIS.

The paper is structured as follows. In the data and methods section, the study area, the data sets of remote sensing observations and in-situ measurements, the different methodological aspects and an error analysis on cloud effects are presented. In the results section, the remote sensed identified sulphur plumes are validated with in-situ measurements of H_2_S and S^0^ found in the literature. The spectral properties of coastal sulphur plumes are derived by inter-comparison with reflectance products of satellite sensors with different spectral and spatial resolutions. Their seasonal and annual cycles are quantified. In the following discussion section, their temporal variability is discussed using other satellite data, including information about the sea surface temperature, winds, and sea level anomaly, as well as in-situ measurements and their derived products which include water mass fraction, currents and dissolved oxygen. Finally, main findings of this work are given in the summary and conclusion section.

## Data and methods

### Satellite data

#### Water-leaving reflectance

The reduced resolution (RR) Level-2 product of water-leaving reflectance (ρw) from the ocean colour sensor MERIS of the ESA satellite ENVISAT are used to study the spectral characteristics of sulphur plumes, in order to detect the coastal sulphur patches and their temporal variability. The data are available in the time period from April 2002 to April 2012 as daily scenes with a spatial resolution of 1 km × 1 km, through the MERIS Catalogue and Inventory (MERCI) system of the ESA. The data of the current processed 3^rd^ version are used. The water-leaving reflectances are continuously validated by the MERIS quality working group. Only their validation results for case 2 waters are relevant as the water masses of the considered study area belong to coastal optical complex waters [36]. For case 2 waters, the official ESA data reprocessing validation report declares relative per cent errors (RPD) of < 12.7% and root mean square errors (RMSE) of < 6.11x10^-3^ in the wavelength range between 412.5 nm and 680.9 nm [37]. The RPD and RMSE are two statistical metrics to measure the uncertainties of water-leaving reflectance in the different MERIS channels (see [38] for definition). The only existing paper related to the Namibian Benguela upwelling area points to RPD smaller than 20.0% [36].

The water-leaving reflectance of MODIS (Moderate Resolution Imaging Spectroradiometer) of the Aqua and Terra platforms, HICO (Hyperspectral Imager for the Coastal Ocean) on the International Space Station and MSI (Multispectral Instrument) of Sentinel-2A (ESA Copernicus Programme) are also used for the investigation of the spectral characteristics of coastal sulphur plumes. The MODIS and HICO data are available from the Ocean Biology Processing Group (https://oceancolor.gsfc.nasa.gov/), and the Sentinel-2A data from the Sentinels Scientific Data Hub (https://scihub.copernicus.eu/). Different spatial resolutions are available depending of the sensor (MODIS: 1 km, 500 m and 250 m, HICO: 90 m, MSI: 10 m, 20 m and 60 m). The channels in the visible spectral range were mostly used (MODIS—10 bands: 412.5 nm to 678.0 nm, HICO—56 bands: 404.1 nm to 719.1 nm every 5.7 nm, MSI—9 bands: 443.0 nm to 865.0 nm). The MODIS Level-2 product of remote sensing reflectance (Rrs) was transformed to the water-leaving reflectance with the standard equation ρw = π*Rrs. For the work presented in this paper, the Level-1 products of HICO and MSI were processed to the Level-2 products of water-leaving reflectance with the software packages of SeaDAS (https://seadas.gsfc.nasa.gov/) and ACOLITE [39], respectively.

MODIS reflectances agree very well with in-situ data at 488 nm, 531 nm and 555 nm, but they are too low at 412 nm caused by wrong estimation of aerosol absorption [40–42]. Up to now only few validation studies exist for HICO and MSI. The reflectances of the HICO sensor between 560 nm and 610 nm correspond very well with in-situ measurements performed in the coastal area near Northport, New York [43]. In this wavelength range, the RPD's are lower than 2%. Higher deviations were observed outside this wavelength range of up to 30% around 500 nm. A regional analysis in the Lake Mulwala (Victoria, Australia) demonstrated that the deviations of MSI sensor to the OLI (Operational Land Imager) sensor on Landsat-8 are smaller than 15% for 560 nm, 665 nm, 740 nm, 842 nm and 865 nm but are higher for 443 nm (65%), 490 nm (25%), and 783 nm (27%) [39].

Quasi-true colour images of MERIS, MODIS, HICO and MSI derived from reflectance channels in the red, green and blue (RGB) were produced. These images are used for validation and comparison of remotely sensed identified sulphur plumes with in-situ measurements of H_2_S and S^0^ in the studied area.

#### Cloud fraction

The daily cloud fraction of MODIS Terra is available with a spatial resolution of 1°, through the Goddard Earth Sciences Data and Information Services Center (GES-DISC). In the coastal region of the study area, the seasonal and annual area-averaged cloud fractions were calculated with the Interactive Online Visualization and Analysis Infrastructure (GIOVANNI, http://daac.gsfc.nasa.gov). We use these cloud fractions to investigate the impact of clouds on the sulphur plume time series.

#### Sea surface temperature

The sea surface temperature (SST) of MODIS Aqua in the nearest coastal area is used as a proxy for the upwelling intensity. This approach will be justified in the discussion section by comparing our coastal MODIS SST with the IBU (Intense Benguela Upwelling) index of Hagen et al. [44] which represents another proxy for the upwelling intensity. In their investigations, water mass analysis suggests that the 13°C SST isotherm can be used to describe the offshore boundary of intense upwelling processes. In using this 13°C criterion, they determine the total area of IBU by summation of all pixels between the 13°C isotherm and the coast line. Especially, this 13°C threshold is used in our studies for the characterization of the main upwelling season with the highest upwelling intensity. For the calculation of our proxy for the upwelling intensity, the 4 μm SST product of MODIS Aqua measured at night time is chosen to minimize the effects of solar radiation and water vapour. The monthly SST data with a spatial resolution of 4 km are available through the systems of GES-DISC and GIOVANNI. The nearest coastal area limited by the two main upwelling cells of Walvis Bay and Lüderitz [45] is taken into account for the calculation of the upwelling intensity because the sulphur plumes are mainly observed in these regions [46]. To do so, the coastal SST values are averaged over the box from 23°S-28°S and 14.3°E-15.6°E.

M*oreover*, SST data of TMI (TRMM Microwave Imager) with a spatial resolution of 25 km are applied to check the MODIS SST [47]. The monthly TMI data are available from the Remote Sensing Systems (http://www.remss.com/). Positive SST annual anomalies of MODIS and TMI are also used as proxies for the intrusion of oxygen depleted water into the Namibian shelf area [48–50]. For more details, please see the section about the discussion of seasonal and annual variability.

The SST bias of MODIS Aqua is in the range of 0.07°C and 0.79°C [51–54]. The bias mainly depends on the aerosol optical depth, wind speed and satellite zenith angle [54]. During an in-situ validation campaign using data sets of TAO/TRITON (Tropical Atmosphere Ocean/Triangle Trans-Ocean Buoy Network) and PIRATA (Pilot Research Moored Array in the Tropical Atlantic) a mean bias of -0.07°C and a standard deviation of 0.57°C were derived for the TMI SST [55].

#### Wind speed and pseudo wind stress

The wind products of TMI, QuikScat (Quick Scatterometer), ASCAT (Advanced Scatterometer) and CCMP (Cross-Calibrated Multi-Platform) are produced by the Remote Sensing Systems (http://www.remss.com/). The satellite products of TMI, QuikScat and ASCAT are Level-2 products. The CCMP Level-3 products are merged from satellite, moored buoy, and model wind data [56]. The wind speed products as well as the v- and u-components of pseudo wind stress at 10 m high with a spatial resolution of 25 km are used to study their impact on the variability of sulphur plumes. Different satellite wind products are used due to their different available time series and variables over the studied period. For example, QuikScat and ASCAT do not overlap the full study period of 2002 to 2012 and TMI provides only the wind speed. For the work presented in this paper, the wind data of the nearest coastal area limited by the upwelling cells of Walvis Bay and Lüderitz are used as for the SST data. However, the box is extended due to the reduced spatial resolution of the wind data compared to the SST data. To do so, the wind values are averaged over the box from 23°S-28°S and 13.3°E-15.6°E.

The RMSE in wind speeds of TMI, QuikScat, ASCAT, and CCMP are 0.8–1.6 m s^-1^ [57, 58], 1.0–1.7 m s^-1^ [59–61], 0.1 m s^-1^ [62] and 0.8 m s^-1^ [56], respectively.

#### Sea level anomalies

The sea level anomalies (SLA) from the gridded AVISO^+^ product (Archiving, Validation, and Interpretation of Satellite Oceanographic project) are used in this paper. We take the merged product made with the altimetry sensors (T/P, ERS, ENVISAT, Jason) available for the 2002–2012 time period. The SSALTO/DUACS product (Ssalto multi-mission ground segment/Data Unification and Altimeter Combination System) distributed by CMEMS (European Copernicus Marine Environment Monitoring Service) is used with a daily frequency on a 1/4° Cartesian grid (http://www.aviso.altimetry.fr/). Spatial averaged SLA values between 18°S and 19°S within the 1° width coastal band are used as proxies for the arrival of coastal trapped waves into the study area. More details are given in the section about the discussion of seasonal and annual variability. The SLA bias of the SSALTO/DUACS product is less than ±1 cm.

### In-situ measurements

**Fig 1:**
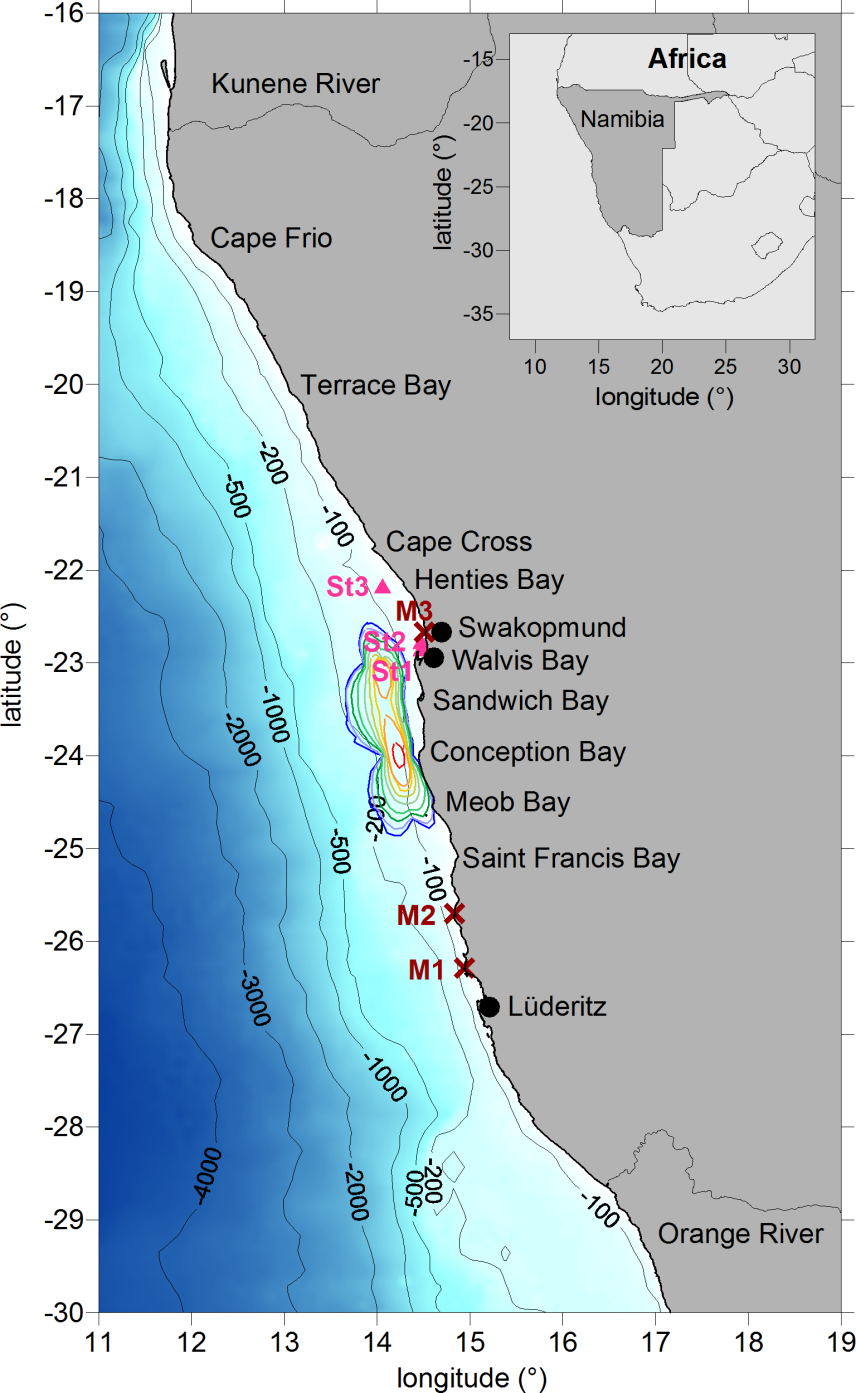
Study area. The figure includes the bathymetry, important Namibian bights and towns as well as rivers (Kunene, Orange). The Namibian shelf is given as 200 m isobaths. The crosses M1 to M3 mark the locations of in-situ measurements of H_2_S and S^0^ which were collected in the upper water column in 2002 [16]. The colored isolines represent the in-situ measurements of bottom water concentrations of H_2_S for the year 2004 [10]. The concentrations were given from blue to red in steps of 0.5, 1, 2, 4, 6, 8, 10, 20 and 30 μM. The triangles St1 to St3 correspond to the locations of bottom water H_2_S concentrations measured in the years 2002, 2003 and 2004 [24].

In-situ measurements of H_2_S and S^0^ in the water column are used for validation (Fig 1). These measurements are compared with our produced sulphur plume products derived from remotely sensed data. Only few in-situ data are available in the Namibian coastal area over the 2002–2012 period. In the year 2002, Weeks et al. [16] measured eight times the concentrations of H_2_S (see the locations of stations M1 to M3 in Fig 1) in the surface water layer during satellite overflights. However, the concentrations of S^0^ (first 10 m depth) were measured only one time during sulphur plumes. Lavik et al. [10] measured the bottom water concentration of H_2_S over a large area of about 7000 km^2^ in January 2004 (colored isolines in Fig 1), a year with high activity of coastal sulphur plumes [46]. Bottom water concentrations of H_2_S on the Namibian shelf were also measured by Brüchert et al. [24]. Hydrogen sulphide was detected in the coastal inshore bottom waters near the Walvis Bay area in March 2002, August 2003 and May 2004 (see the locations of stations St1 and St2 in Fig 1) but near the Henties Bay region only in September 2003 and March 2004 (station St3 in Fig 1).

Other in-situ measurements and their derived products (water mass fraction, dissolved oxygen, currents) of different authors as well as the Commonwealth Scientific and Industrial Research Organisation (CSIRO) Atlas (CARS) for oxygen climatology were used for the discussion of seasonal and annual variability of sulphur plumes.

### Methods to determine sulphur plume size and intensity

The study area covers the Namibian shelf between the Kunene and Orange rivers between 18°S-28°S (Fig 1). The sulphur plumes cannot be monitored systematically by field observations, marine expeditions, deployments or float and glider systems because of their sporadic occurrence and coastal location offshore of Namibian desert regions. However, their milky turquoise patches at the sea surface can be observed with remote sensing by ocean colour satellite sensors [23, 46]. In the current paper, the coastal sulphur plumes are identified in RR-MERIS-Level-2 data of water-leaving reflectance with an identification algorithm using differences in the spectral reflectance of various optical water masses such as algae blooms, river plumes and sulphur events. The basic method introduced by Ohde et al. [46] is improved and their implementation in the software package of the interactive data language (IDL) is updated in this paper. We explain in the following our improved methodology:

1. The full time period of the MERIS sensor from April 2002 to April 2012 are processed.
2. The study area is extended to the coastal area between 18°S and 28°S.
3. The water-leaving reflectance of MERIS of the current 3^rd^ version is used.
4. Daily satellite swaths are combined to single MERIS scenes if different swaths overlaying the study area for the same day. The scenes are gridded to the same Mercator projection by the ESA-Sentinel Application Platform (SNAP).
5. The four conditions for the detection of sulphur plumes defined in Ohde et al. [46] are adapted to the 3^rd^ reprocessing by the definition of new threshold values using the full MERIS data set. The first condition ρw (412.5 nm) < ρw (442.4 nm) are not changed. The second (ratio ρw (664.6 nm) to ρw (680.9 nm)) and third (peak value of ρw (559.6 nm)) conditions are adapted only slightly. Now the threshold values of 1.0054 (old: 1.0146) and 0.0400 (old: 0.0479) are applied. The fourth condition is modified using the full MERIS data set of 2002 to 2012. Now the slope between ρw (559.6 nm) and ρw (619.6 nm) is used to better exclude pixels associated with Orange river plume and resuspended matter in near-shore coastal areas.
6. The size of sulphur plumes in square kilometers and their remotely sensed intensity using ρw (559.6 nm) are determined.

Two new methods are applied to characterize the temporal variability of sulphur plumes. In each of these methods, a spatial averaged Level-2 product (basic product) is derived which is the basis for the calculation of temporal averaged Level-3 products:

1. In the first method, the daily size of sulphur patches in square kilometers (basic product 1) is determined in each of the 3433 available MERIS scenes. From the daily values, monthly integrals of size are calculated (sum of the daily integrals over the month). No statistics based on mean values is derived as the size values of sulphur patches are not normal distributed. Also no median values are determined to avoid corruptions by zero values coming from days with no sulphur events. Furthermore, integrals of size quantify the plume extensions better than means and medians. A seasonal and annual variability of the size is derived using the monthly integrals over the 2002–2012 time period. To do so, integral values of size are deduced for each month from January to December and for each year. Days without sulphur plumes do not contribute to these integral values.
2. In the second method, the daily median value of remotely sensed intensity (basic product 2) is determined for all days of the 2002–2012 time period. This variable is dimensionless. The water-leaving reflectance of the special waveband at 559.6 nm is used as all sulphur spectra of coastal events are characterized by a reflectance maximum at this wavelength (details in the validation section). A statistics based on median intensities is chosen for the determination of their seasonal and annual variability because no normal distribution is found for the water-leaving reflectance distribution (details in the validation section). The seasonal and annual variability of intensities are derived using the monthly intensities (see seasonal and annual variability section below).

The advantage of this new approach is the investigation of the temporal variability of sulphur plumes with two independent methods based on size and intensity of sulphur plumes. This new approach allows also to check the robustness and consistency of the results of both methods as well as to evaluate the cloud influence.

**Fig 2:**
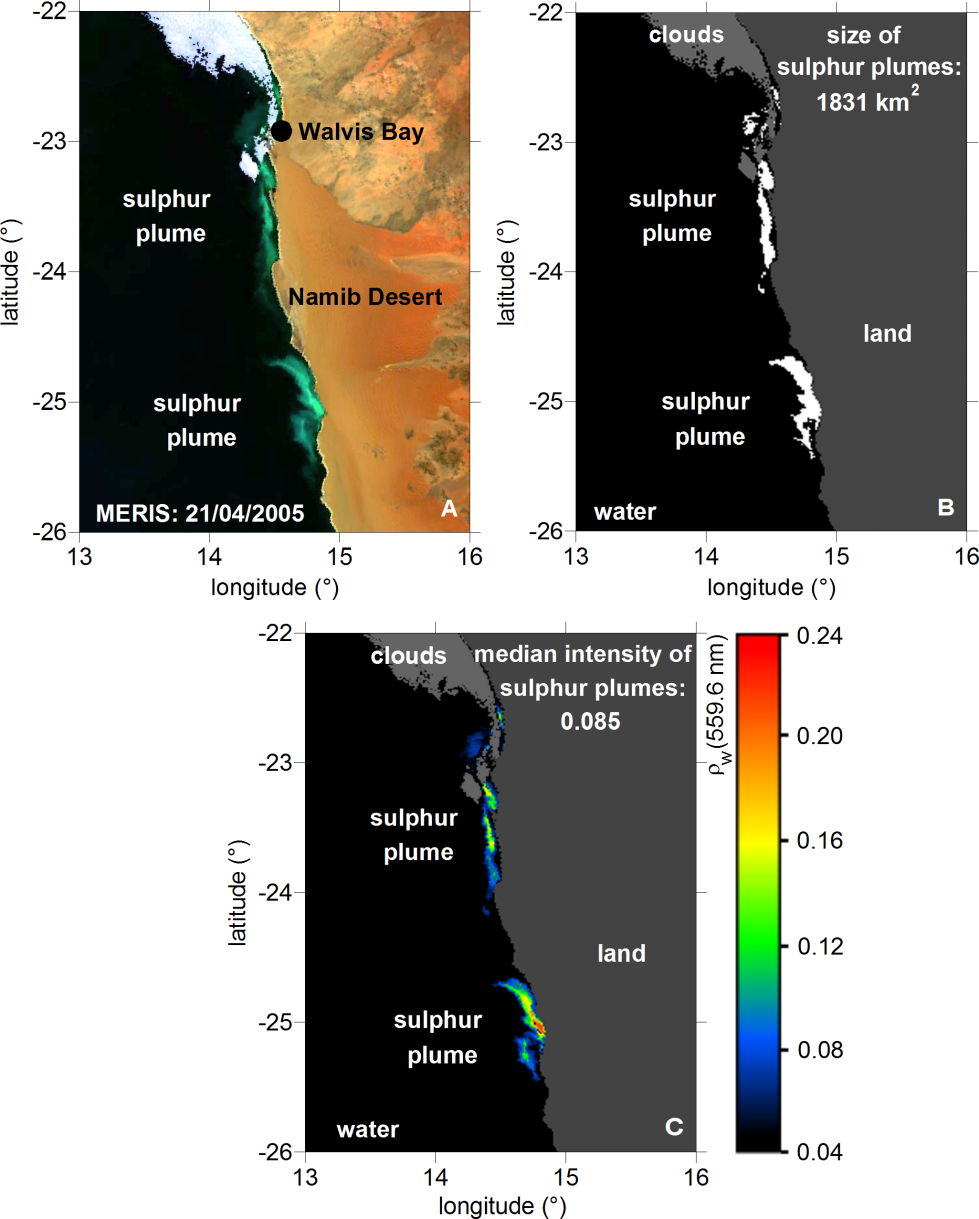
Example for the detection of sulphur plumes. (A): Sulphur plumes as MERIS RGB image in quasi-true colours on 21 April 2005. (B): Identified sulphur pixels including the size of the sulphur patches on this day corresponding to basic product 1 (white pixels in Fig 2B). (C): Sulphur pixel intensities based on median intensity on this day corresponding to basic product 2 (colored pixels in Fig 2C).

Fig 2 illustrates an example for the detection of sulphur pixels in the MERIS scene on 21 April 2005. The identification algorithm delivers the size of the sulphur patches (white pixels in Fig 2B) which corresponds to the basic product 1 of the first method. The comparison of the RGB image of Fig 2A with the identified sulphur patches of Fig 2B demonstrates the conformity of the areas of milky turquoise discolorations with the identified sulphur pixels. Fig 2C corresponds to the daily values of remotely sensed intensity of sulphur pixels. The daily median value represents the basic product 2 of the second method.

### Methods for seasonal and annual variability

The variability of sulphur plumes is investigated in relation to local and remote-driven environmental conditions. For this purpose, the seasonal as well as the annual variability of sulphur plume size and intensity are determined and compared with those of the environmental variables. The seasonal variability is determined in the same way for the sulphur plume intensity and environmental variables. The seasonal variability is estimated with a monthly climatology using the data over the 2002–2012 time period. Values of minimum, maximum, median, 25^th^ percentile (1^st^ quartile) and 75^th^ percentile (3^rd^ quartile) are calculated for each month from January to December to produce box and whisker plots. For the annual variability of the sulphur plume intensity and environmental variables, anomalies are estimated with the differences between the yearly means and the global mean of years 2002 to 2012. It is not always possible to calculate a monthly climatology as well as box and whisker plots for each of the environmental variables because of the small number of available in-situ data (e.g. H_2_S, S^0^**)** and gaps in their time series (e.g. currents). In such cases, the discussion about the relations between in-situ measurements and sulphur events is based on a qualitative analysis.

### Error analysis

The basic Level-2 products of size (product 1) and remotely sensed intensity (product 2) as well as all derived Level-3 products (e.g. seasonal climatology) are limited in their accuracy. Misinterpretations in the determination of the sulphur patches size can appear at the borders between sulphur plumes and other optical water bodies. The error depends on the size of detected areas. For the product 1, the errors are of the order of 10% for 1000 km^2^ and of 5% for 10000 km^2^. The current validation of the water-leaving reflectance at 559.6 nm delivers an error of the order of 6.3% for the product 2 [37].

The influence of clouds on the determination of the seasonal and annual variability of sulphur plumes is investigated using the MODIS product of cloud fraction. The mean area-averaged cloud fraction and their standard deviation are calculated for a monthly climatology from January to December and for each year over the 2002–2012 time period (S1 Document). A statistics based on mean values is derived as the values of cloud fraction are nearly normal distributed. As supplementary criterion for the impact of clouds, the number of days in percentage where the cloud fraction exceeds a certain defined threshold of 2/3 for 3 consecutive days was derived (S1 Document). For these days, it is assumed that the sulphur plumes cannot be detected.

A pronounced seasonal cycle of cloud fraction is observed (cf. values of Mcfm in S1 Document). The cloud coverage is highest between December and February and lowest between May and July. Also, there are strong monthly variations for the number of sulphur plumes which are probably not detectable in the study area (cf. values of Ncfm3d in % in S1 Document). Up to 25% of the sulphur plumes could be overlooked in February but only 2% in June. This seasonal cycle may have an impact on the sulphur seasonal cycle. However, the direct comparison between the seasonal cycles of sulphur plumes and clouds in the section about the seasonal variability will demonstrate the opposite. The corresponding investigations for the influence of clouds on the determination of the annual variability of sulphur plumes are summarized in the second part of the S1 Document. Nearly the same mean area-averaged cloud fraction and standard deviation are derived for all considered years. It means, the cloud conditions between the years are comparable with no exceptional deviations. Thus the clouds have the same impact on each year of the annual sulphur time series. Furthermore, the sulphur patches are detected if a certain percentage of pixels in the study area were cloud-free at least on one day within the mean lifetime. Only 7 to 16% of the sulphur plumes are probably not detected if a mean lifetime of sulphur plumes of 3 days and a cloud fraction threshold of 2/3 are assumed. This result documents again the low influence of clouds on the determination of the annual variability of sulphur plumes. The product of remotely sensed intensity is more independent from the cloud impact than the product of size because only few pixels are necessary for the determination of the sulphur plume intensity as compared to the sulphur plume size.

## Results

### Validation and spectral properties of sulphur plumes

The validation of sulphur spectra of the MERIS sensor is not directly performed because no in-situ sulphur spectra were measured in the Namibian coastal area at the same time as MERIS overflights. However, some in-situ measurements of H_2_S and S^0^ are available for MODIS overflights. Furthermore, inter-comparisons with Level-2 reflectance products of satellite sensors of different spectral and spatial resolutions like MODIS, HICO and MSI are realized.

**Fig 3:**
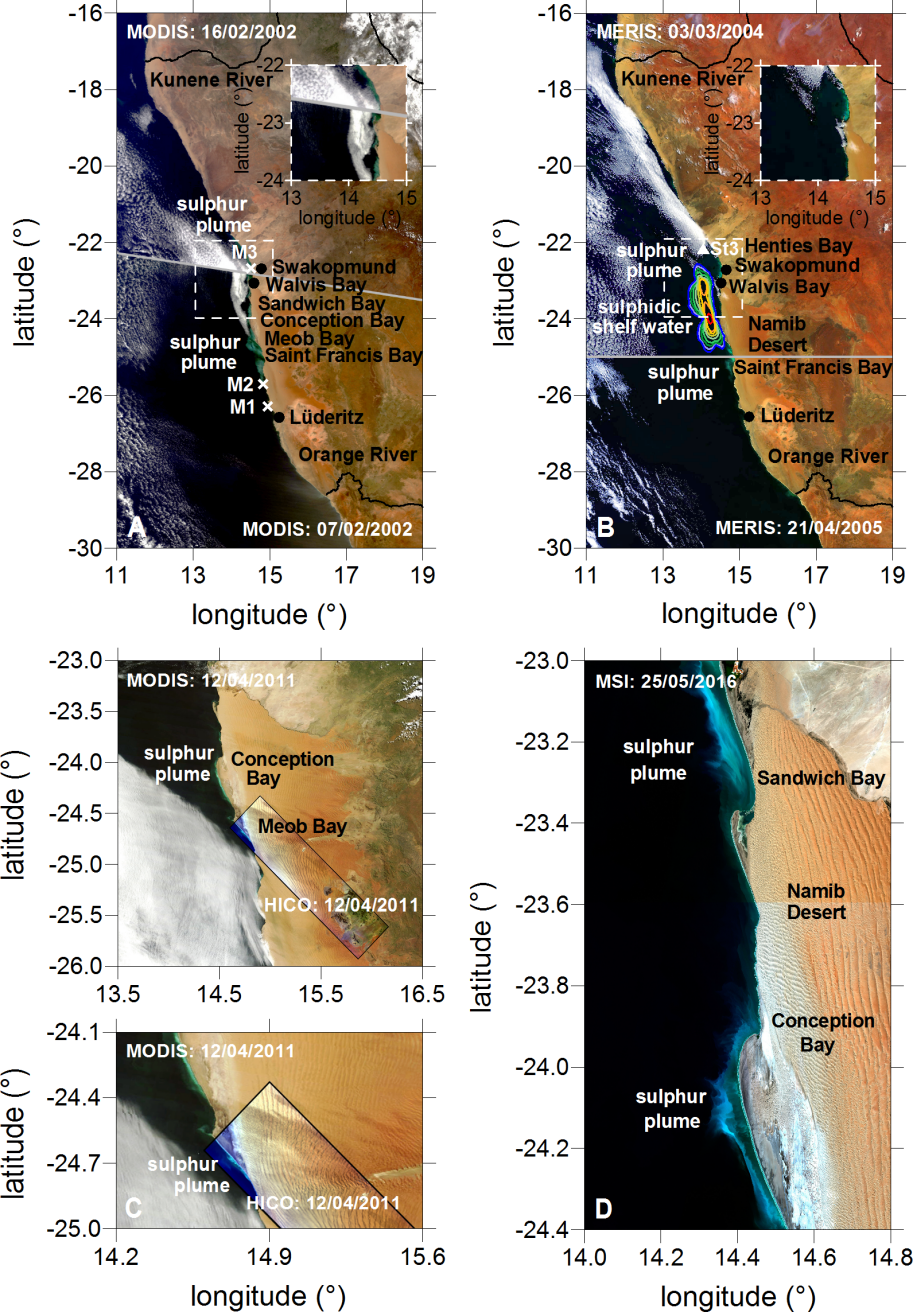
Sulphur plumes seen from satellites. (A) and (B): Each of the mosaics of MODIS and MERIS is composed from two RGB images. The grey-line is the limit between them. The locations of in-situ measurements which were performed in the upper surface water layer during sulphur plumes seen from MODIS are marked with M1 to M3 [16]. The measurements of Lavik et al. [10] are marked with the same isolines as in Fig 1. The triangle St3 corresponds to the measurements of Brüchert et al. [24]. The dashed lines represent zooms in specific areas. (C): The MODIS scene is overlayed by the only available HICO scene. (D): The spatial high resolution MSI scene (10 m) characterizes the fine structures of sulphur plumes.

Only five in-situ campaigns of Weeks et al. [16] are usable for validation because H_2_S and S^0^ were measured in the upper water column during MODIS overflights with nearly cloud free conditions. For example, two of the five available MODIS scenes are given in Fig 3A. Both scenes from 7 and 16 February 2002 are arranged to one mosaic to show the sulphur patches. Lavik et al. [10] measured high bottom water concentrations of H_2_S in January 2004. The spatial distribution of these sulphidic shelf waters is given in Fig 3B. The bottom-water concentrations of sulphide were between 0.5 μM (blue line) and 30 μM (red line). In March 2004, Brüchert et al. [24] determined about 2 μM in the bottom water layer near the Henties Bay (station St3 in Fig 3B). Coastal surface sulphur plumes were detected in the MERIS scene on 3 March 2004 (Fig 3B).

**Fig 4:**
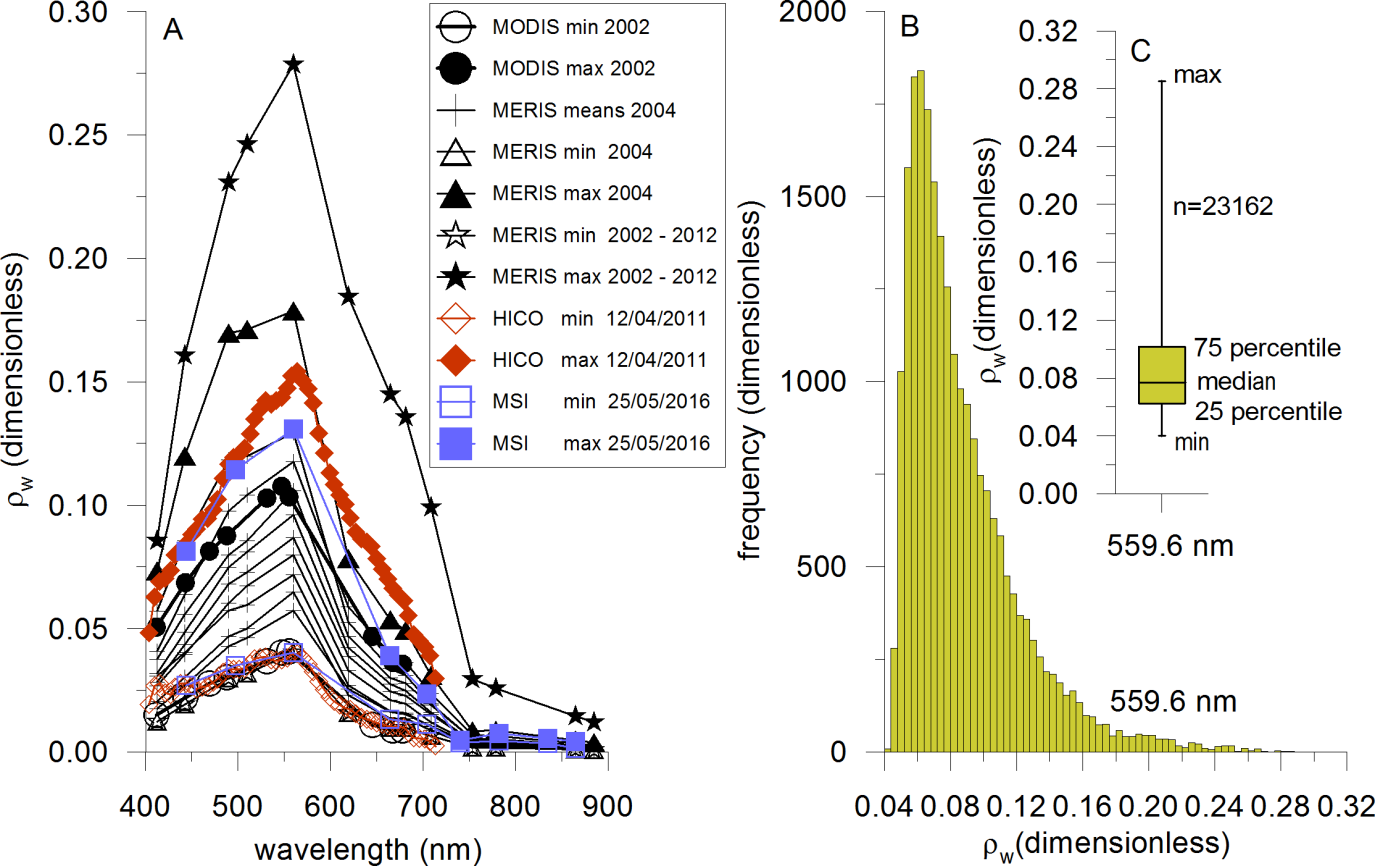
Spectral properties of sulphur plumes and statistics. (A): Comparison of sulphur spectra of different satellite sensors like MERIS, MODIS, HICO and MSI. The MODIS spectra were collected at the same time of in-situ measurements of Weeks et al., [16]. The MERIS spectra represent mean values of the main sulphur events in 2004. The minimum and maximum spectra of MERIS were determined from all sulphur events of year 2004 as well as of years 2002 to 2012. The spectral high resolution curves of HICO were determined from the only available scene from 12 April 2011. The MSI spectra were derived from a scene of 25 May 2016. (B): Density distribution of reflectance values of MERIS at 559.6 nm determined from all sulphur pixels in the 2002–2012 time period. (C): Minimum, median, maximum, 25^th^ and 75^th^ percentiles of all sulphur reflectance values in the studied area at 559.6 nm.

The combination of in-situ measurements of Weeks et al. [16], Lavik et al. [10] and Brüchert et al. [24] with the remote sensing products of MODIS and MERIS demonstrates the close relationships between H_2_S, S^0^ and the milky turquoise discolorations in the Namibian coastal area, which is the basis for the investigation. During different in-situ campaigns high concentrations of H_2_S were measured by Weeks et al. [16] on 17 to 18 March 2001, 26 March to 4 April 2001, 7 to 8 February 2002, 14 to 15 February 2002 and 3 to 4 March 2002, and of S^0^ on 14 to 15 February 2002 in the upper water column. The minimum and maximum sulphur spectra which were determined from all MODIS sulphur pixels during these in-situ campaigns are characterized by specific spectral signatures (Fig 4A, S1 Table). Very high reflectance values in the entire spectral range due to the high backscattering of small sulphur particles (S^0^) are observed. The reflectance of the plumes increases and decreases monotonously in the short and long wavelength ranges, respectively, without any indication for chlorophyll absorption [46]. The minimum and maximum spectra of MERIS derived from all detected sulphur pixels between the years 2002 and 2012 as well as the mean spectra of the main events of year 2004 are also displayed in Fig 4A. As explained in the method section, the sulphur plumes can be clearly distinguished from other optical water masses (see also the supporting information in the S2 Document). The spectral shape of the minimum spectrum of MERIS matches very well the minimum spectrum of MODIS (Fig 4A). The mean spectra of the main sulphur events of MERIS of year 2004 correspond with the spectral shape of the maximum spectrum of MODIS. However, the maximum spectrum of MERIS of the whole considered time period is higher than that of MODIS. Indeed it presents the highest intensity in the 10-years period between 2002 to 2012 compared to the smaller period of 2001 to 2002 of MODIS. Up to 28.5% of the light at the wavelength of 559.6 nm can be backscattered by sulphur plumes (Fig 4A). All coastal sulphur spectra of MERIS are characterized by a maximum at this wavelength confirming the usage of the corresponding MERIS waveband for the characterization of the sulphur plume intensity (section about the methods to determine sulphur plume size and intensity, basic product 2). The corresponding reflectance values of all detected sulphur spectra (23162 values) are plotted in the histogram in Fig 4B (S2 Table). No normal distribution was found verifying the calculation of median values for the monthly, annual and seasonal intensities of sulphur plumes (section about the methods to determine sulphur plume size and intensity, Level-3 products). The statistics of reflectance values at the wavelength of 559.6 nm is given in Fig 4C (S2 Table). The minimum and maximum values are ρw (559.6 nm) = 0.040 and ρw (559.6 nm) = 0.285, respectively. A median value of 0.077 was found. Only 25% of the values are lower and greater than 0.062 and 0.101, respectively. Unfortunately, no investigation about the relation between the S^0^ concentration and the intensity of sulphur plumes can be performed because no in-situ measurements of S^0^ are available at the same time of MERIS overflights. However, a rough approximation is possible with MODIS data. In-situ measurements of Weeks et al. [16] on 14 to 15 February 2002 delivered S^0^ concentrations between 773 nM and 1802 nM in the surface water during MODIS overflights with cloudfree conditions. These concentrations correspond to MODIS intensities between 0.045 and 0.081. This suggests that sulphur events with S^0^ concentrations lower than 631 nM can not be detected with our algorithm if we assume a linear relation between sulphur plume intensity and S^0^ concentration as well as if we take into account the lower limit of 0.04 (see section “Methods to determine sulphur plume size and intensity”).

Additionally, the sulphur spectra of HICO (Fig 3C) and MSI (Fig 3D) are included in Fig 4A to investigate the spectral properties in detail. The comparison of MERIS with HICO and MSI demonstrates again the good agreement of spectral shapes at low intensities. Only deviations in the blue spectral range are observed for the HICO sensor caused by the absence of an on-board calibrator [63]. The maximum spectrum of MERIS is higher because the data set of this sensor is over a long time period (10 years). Only one scene of HICO (12 April 2011, Fig 3C) and only two scenes of MSI (25 May 2016 as example, Fig 3D) are available for the study of sulphur plumes due to their low repeat cycles coupled with the sporadic occurrence of sulphur events. The maximum of HICO sulphur spectrum is observed at a wavelength of 564.5 nm (Fig 4A). It is really near the MERIS maximum at 559.6 nm which emphasizes again the good position of this waveband for the characterization of the sulphur intensity. The spectral high resolution HICO data illustrates with more details the sulphur spectrum than the MERIS data, for instance the second spectral peak around 530.1 nm (Fig 4A). However, this spectral information could not be used for the identification algorithm using the MERIS sensor due to its limited band number.

### Seasonal and annual variability of size and intensity of sulphur plumes

#### Seasonal variability

**Fig 5:**
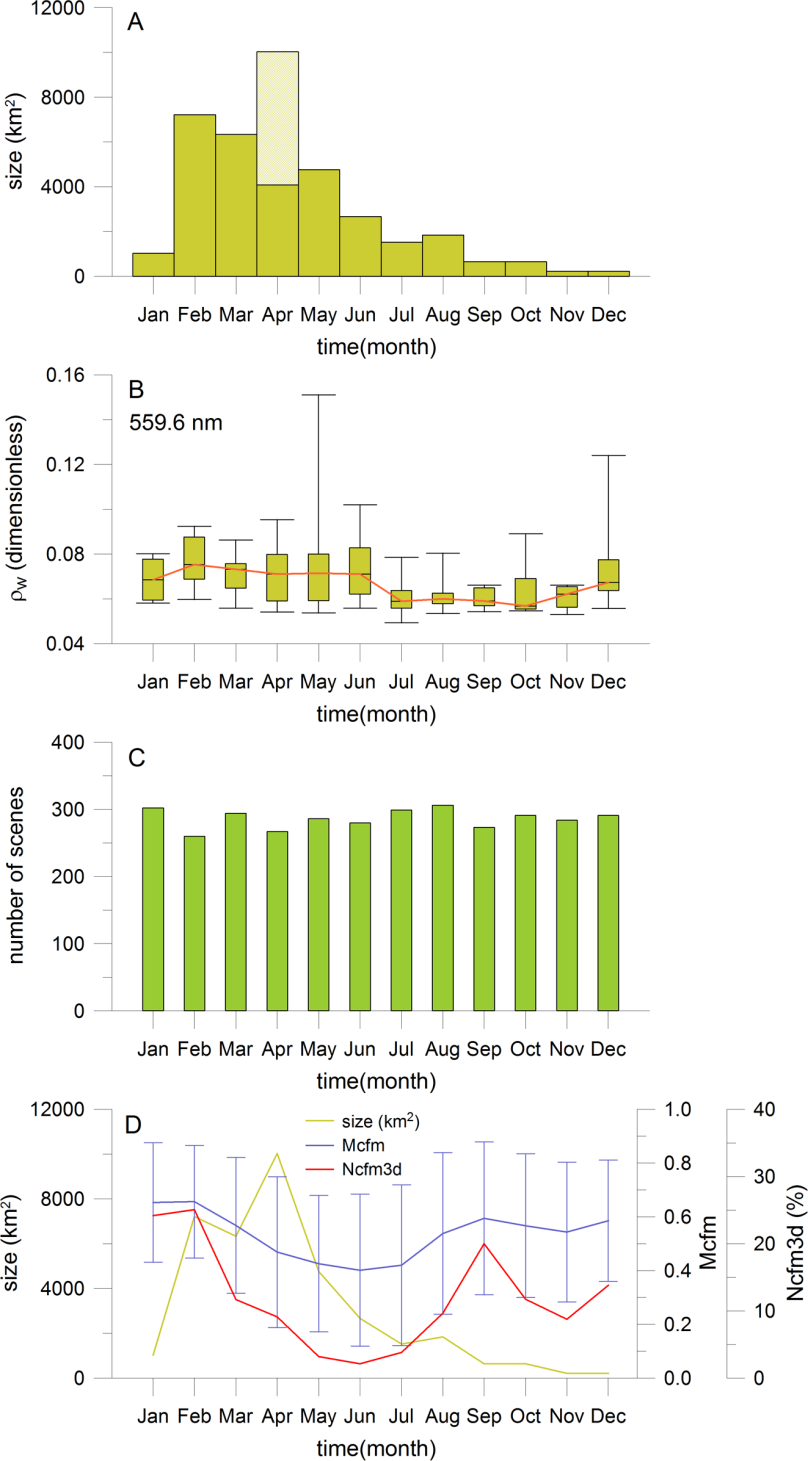
Seasonal variability of sulphur plumes. (A): Seasonal variability of sulphur plume size derived from all MERIS data of years 2002 to 2012. The light yellow bar in April represents the sulphur plume size including the strong events of year 2005. (B): Seasonal variability of the sulphur plume intensity with monthly minimum, maximum, median, 25^th^ and 75^th^ percentiles. (C): Number of MERIS scenes available for each month. (D): Sulphur plume size, mean area-averaged cloud fraction including the standard deviation (Mcfm), number of days in percentage where the cloud fraction is above 2/3 on 3 consecutive days (Ncfm3d in %).

The seasonal climatology of size and intensity of coastal sulphur plumes over the study area is presented in Fig 5A and 5B (S3 Table). An apparent seasonal cycle in their size is observed with pronounced main and off-seasons (Fig 5A). The second largest size of 7217 km^2^ is detected in February but special strong events like the sulphur plumes in April 2005 shifts the maximum to April (light yellow bar with 10027 km^2^ in Fig 5A). Then the size continuously decreases up to November and December with minimum values lower than 221 km^2^. The seasonal cycle of intensity of coastal sulphur plumes is only slightly different (Fig 5B). The highest median intensity is observed in February two months earlier than the size maximum in April. The reflectance values at 559.6 nm of specific events are also high in May and December. The intensities slightly decrease between March and June but are low between July and November.

The results of seasonal variability given in Fig 5A and 5B are very robust because of the high number of available MERIS scenes per month (see Fig 5C, S3 Table: 260 to 300 scenes per month) and the high number of detected sulphur pixels (S3 Table: 136 to 6247 pixel per month). However, this variability may be biased by the clouds (see the section about the error analysis). The impact of clouds is investigated in Fig 5D. A well pronounced seasonal cycle of cloud coverage and a strong monthly variability for the number of probably not detectable sulphur plumes are determined (see curves of variables Mcfm and Ncfm3d in % in Fig 5D; S1 Document). Both results could have an influence on the seasonal variability of sulphur plumes. However, the direct comparison of seasonal cycles of sulphur plumes and cloud coverage does not confirm this assumption. For instance, the cloud coverage and the number of probably not detectable sulphur plumes are very high in January and February. However the second-highest sulphur plume size is found in February. This value should be low and at the same level as the value in January if the clouds influence the signal. Furthermore, the cloud coverage and the number of probably not detectable sulphur plumes continuously decrease from February to June. This should increase the probability for the identification of sulphur pixels in MERIS scenes, however the sulphur plume size mainly decreases with the exception of the April month. As described before, the sulphur plume size in April is increased because of the special strong events in April 2005.

#### Annual variability

**Fig 6:**
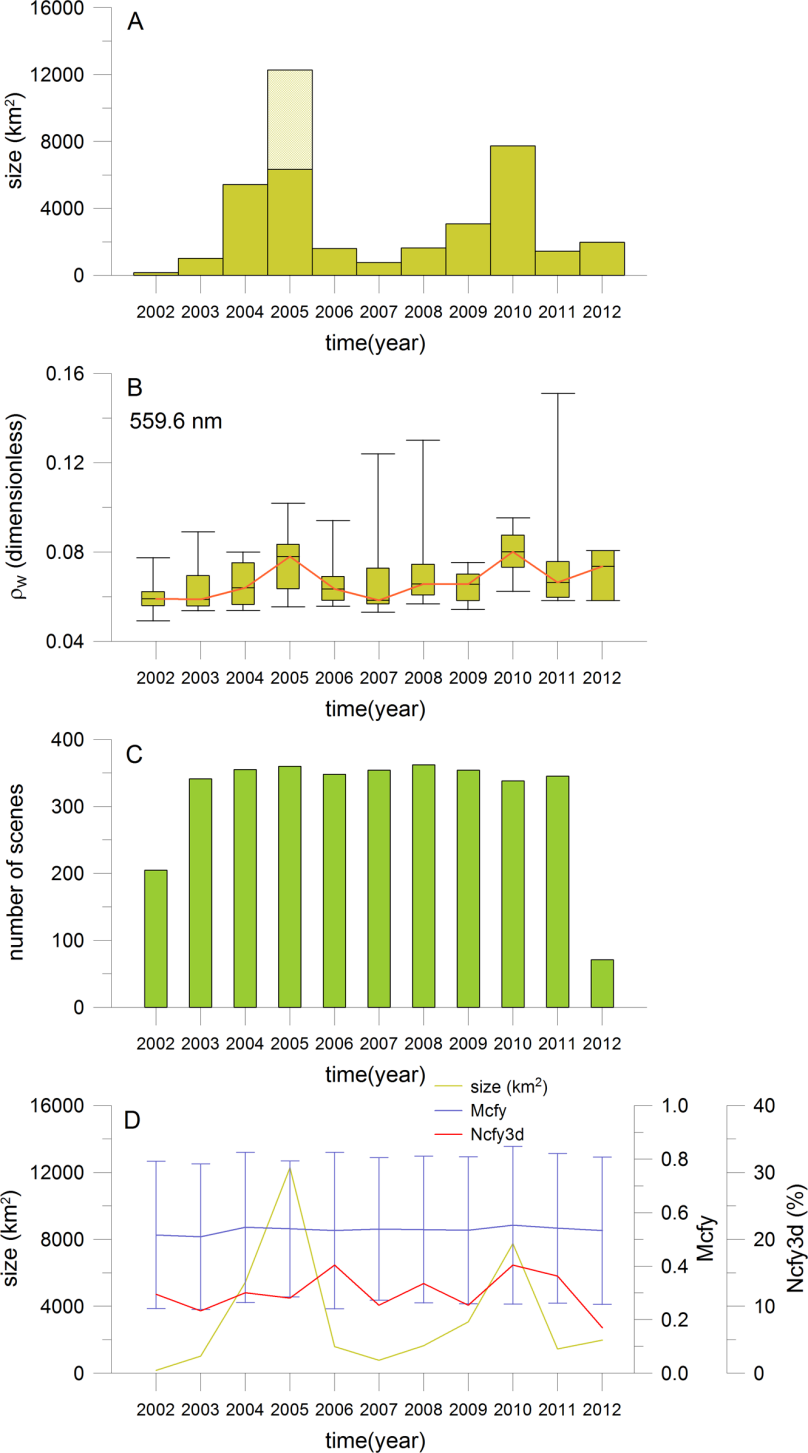
Annual variability of sulphur plumes. Same figure as Fig 5, however for the annual variability.

The annual variability of size and intensity of coastal sulphur plumes was determined from more than 23000 sulphur pixels (Fig 6A and 6B, S4 Table). The annual variability of size is characterized by short phases of very large sulphur plume extensions interrupted by phases of low extensions (Fig 6A). The largest sizes of 12273 km^2^ (6331 km^2^ without the special strong events in April 2005), 7737 km^2^ and 5431 km^2^ are observed in the years 2005, 2010 and 2004, respectively. Low sizes between 165 km^2^ and 3073 km^2^ are found before the year 2004, between the years 2006 and 2009 as well as for the years 2011 and 2012. Nearly the same annual variability is observed for the intensities in terms of reflectance at 559.6 nm (Fig 6B). The highest median intensities are derived for the years 2010, 2005 and 2012. Low median intensities are found before 2005, between 2006 and 2009 as well as in 2011. The maxima of intensities are determined in years with low median intensities such for 2011, 2008 and 2007. The annual variability of sulphur intensity confirms the main findings for the annual variability of sulphur plume size. Exceptions are the years 2004 and 2012. For the year 2004, the sulphur plume size is high but their intensity is low. This is reversed for the year 2012.

The results given in Fig 6A and 6B are very robust because of the high number of used MERIS scenes (see Fig 6C, S4 Table: 71 to 362 scenes per year) and the high number of detected sulphur pixels (S4 Table: 105 to 7646 pixels per year). The sulphur plume size of years 2002 and 2012 may be underestimated because the MERIS sensor was only in operation from April 2002 to April 2012. However, the impact of the missing MERIS scenes should be low because only few sulphur events are observed in the MODIS database between January and March 2002 as well as between May and December 2012. Comparable cloud conditions between the years with no exceptional deviations were already described in the section about the error analysis. This finding and the comparison of the number of not detectable sulphur plumes with the sulphur plume size confirm again the low influence of clouds (see curves of variables Mcfy and Ncfy3d in % in Fig 6D; S1 Document). For instance, the number of probably not detectable sulphur plumes is only between 9 and 12% in the years 2002 to 2005, 2007 and 2009 but the sulphur plume size varies between the lowest and highest detected values. These numbers should be nearly the same if clouds would have an influence.

## Discussion

The seasonal (Fig 5A and 5B) and annual variability (Fig 6A and 6B) of sulphur plumes are discussed in relation to environmental conditions using other satellite data and available in-situ measurements as well as literature. The origin of sulphur plumes in the upper layer is mainly associated with the H_2_S enriched bottom water due to different processes which were explained and summarized in the introduction. Two forcing can maintain the bottom anoxic waters: local forcing associated with wind and remote forcing due to poleward advection of the equatorial water masses by the Angola current and due to poleward coastal trapped waves forced by equatorial Kelvin waves. We will discuss these two kinds of forcing in a first part for the seasonal cycle and in a second part for the annual variability.

### Discussion of seasonal variability of sulphur plumes

#### Relation to local forcing: Winds and upwelling intensity

**Fig 7:**
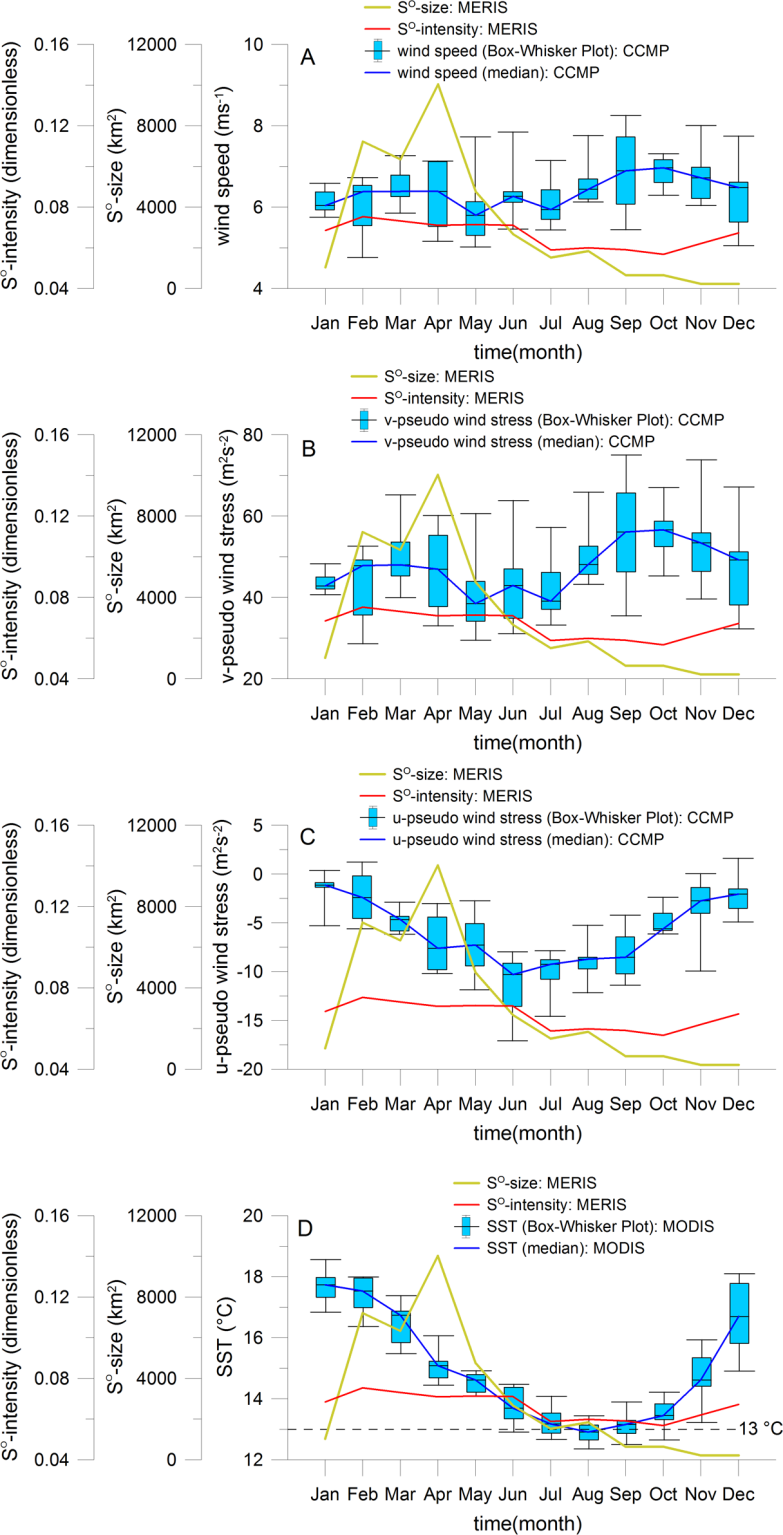
Seasonal variability of sulphur plumes in relation to local forcing. Seasonal climatology of sulphur plume size and intensity, of wind speed at 10 m high of CCMP data set (A), of v-component of pseudo wind stress at 10 m high of CCMP (B), of corresponding u-component of pseudo wind stress (C) and of MODIS sea surface temperature (D). The yellow and red curves in each of these figures correspond to the monthly size and intensity of sulphur plumes derived from all MERIS data of years 2002 to 2012, respectively. For the wind and SST products, Box-Whisker plots with monthly minimum, maximum, median, 25^th^ and 75^th^ percentiles were calculated over the main upwelling cells between Walvis Bay and Lüderitz. The blue curves represent the median values.

Investigations of Weeks et al. [16, 23] demonstrated that the initial surface sulphur signatures overlie the upwelling zone. They often observed the milky-turquoise discolorations at the coastal areas where upwelling-associated cold SST spots were found. As the upwelling activity is directly related to the wind conditions and by that to the coastal SST, we will analyze the wind and the SST seasonal cycles (Fig 7A to 7D).

Indeed the equatorward wind paralleled to the coast (trade winds) can induce a coastal Ekman upwelling [64]. This coastal upwelling affects only a very narrow coastal band of the order of about 30 km to 50 km wide [65]. The wind stress curl also plays a role in the Ekman pumping over the continental slope. However, this effect is not analyzed because the sulphur plumes are mainly influenced by the coastal upwelling due to their zonal extension of about 10–20 km on the continental Namibian shelf [46]. The seasonal wind conditions are given in the Fig 7A to 7C (S5 Table). Our investigations demonstrate that the seasonal cycle of wind speed is mainly driven by the northward alongshore wind component (cf. Fig 7A with Fig 7B, S5 Table). The northward alongshore (given as v-component of pseudo wind stress in Fig 7B, S5 Table) and westwards cross shore (given as u-component of pseudo wind stress in Fig 7C, S5 Table) wind components begin to increase and decrease, respectively, steadily from January to April which increase the wind paralleled to the coast. Consequently, the Ekman transport is increased which enhances the probability of the upwelling of H_2_S enriched bottom water in the Namibian coastal area. Therefore, the sulphur plume activity given as size and intensity peaks during the same time period. The following wind relaxation between May and July causes the reduction of sulphur plume activity. After this period, the wind speed and the corresponding northward alongshore pseudo wind stress increase again which increases slightly the size of sulphur plumes in August. However, this enhancement is much lower than in April.

**Fig 8:**
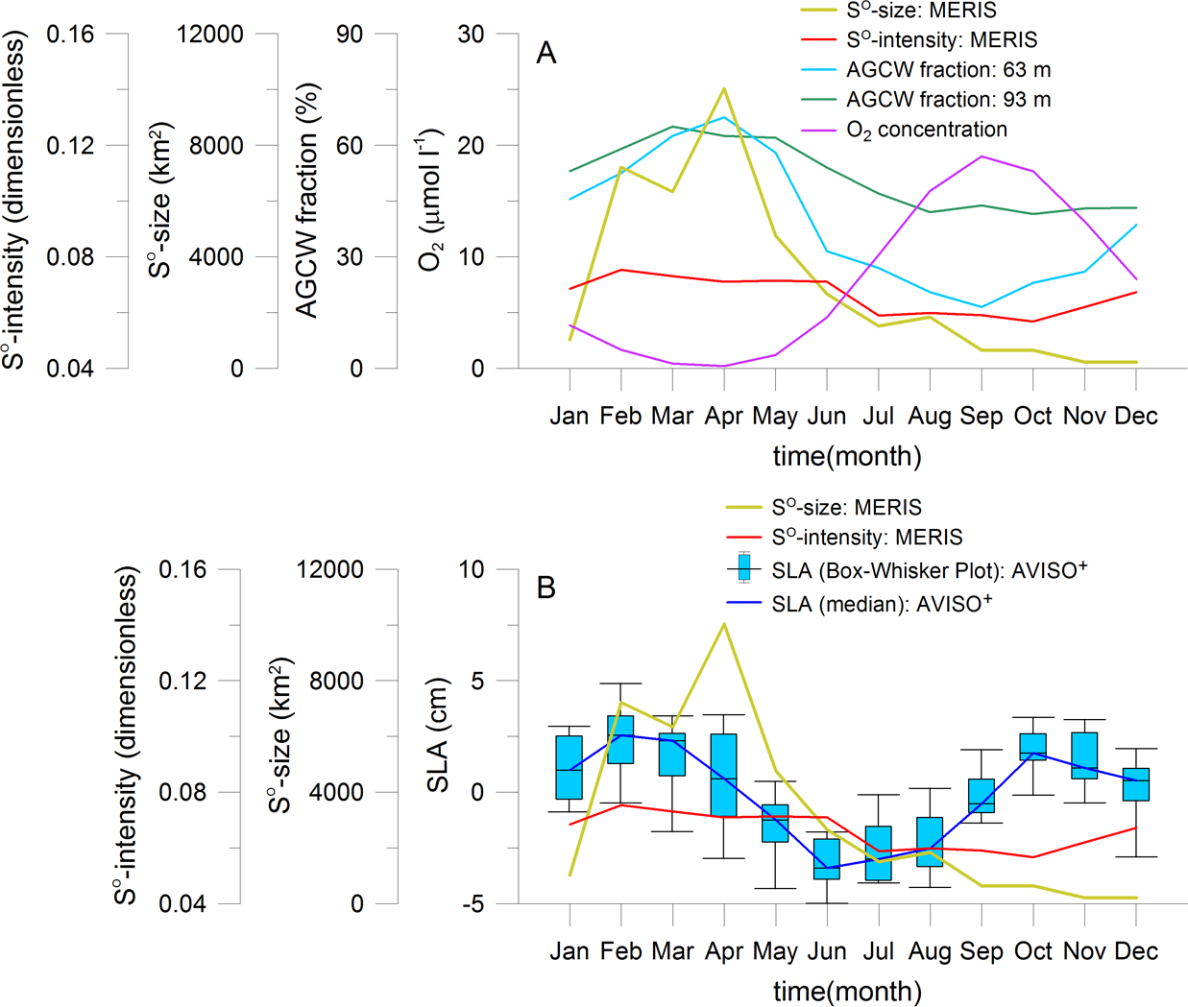
Seasonal variability of sulphur plumes in relation to remote forcing. Seasonal climatology of sulphur plume size and intensity, of AGCW fraction, of CARS oxygen concentration in the bottom water layer (A) and of sea level anomaly between 18°S and 19°S within the 1° width coastal band (B). The yellow and red curves correspond to the monthly size and intensity of sulphur plumes, respectively. The turquoise and green curves represent the AGCW fractions for the depths of 63 m and 93 m, respectively. The pink curve stands for the oxygen concentration. Box-Whisker plots with monthly minimum, maximum, median, 25^th^ and 75^th^ percentiles can be calculated only for the SLA product. The calculation is not performed for AGCW fraction and oxygen concentration because not the full data sets are available. The blue curves represent the median values.

As we will justify in the following that the upwelling intensity can be approximated by the coastal MODIS SST conditions, we will examine the seasonal cycle of sulphur plumes in relation to main upwelling intensity (Fig 7D, S5 Table). The 13°C threshold defined by Hagen et al. [44] characterizing the main upwelling season with the highest upwelling intensity [44] is just reached in June up to October (but July to September within the 25^th^ and 75^th^ percentiles) with a maximum in August (Fig 7D). This result corresponds very well with the IBU (Intense Benguela Upwelling) index (another proxy for the upwelling intensity) describing the total area of cold water between the Namibian coast and the 13°C isotherm position [44]. With this IBU, the main season of cold surface water was observed by Hagen et al. [44] between July and September during the austral winter. This cold surface water peaks in August with an area of about 30000 km^2^. The temperature seasonal cycle derived by Louw et al. [66] using in-situ temperatures at different depths of time period from 2001 to 2011 is also very similar to the SST cycle in Fig 7D. However, there are shifts of two months and one month in the maximum and minimum temperatures, respectively, due to the northern location of in-situ measurements (10 NM station off Walvis Bay at 23°S). The comparisons with the results of Hagen et al. [44] and Louw et al. [66] point out that the coastal MODIS SST represents a good proxy for the upwelling intensity in our study area. At the beginning of the seasonal SST cycle the size and intensity of sulphur plumes continuously increase with the enhancement in the upwelling intensity (Fig 7D). However, the main season for sulphur plume size and intensity (February to May) in the NBUS does not correspond to the main season for the upwelling intensity (July to September). This is not a contradiction because the pool of H_2_S enriched bottom water is full at the beginning of the seasonal cycle before the main upwelling season. The pool is then continuously emptied and is probably dissipated during the main season of the upwelling intensity. Measurements of the H_2_S concentration in the bottom water layer by Brüchert et al., [24] support this assumption because the H_2_S is more often available in austral autumn than in other seasons. Indeed over the continental shelf, the mixing is very high during the onset of the main upwelling in austral winter and so the water column is more oxygenated (see our Fig 8A) which could disrupt the formation of H_2_S. However, the variability of sulphur plumes may not be only influenced by the local effects of wind-driven upwelling but also by remote-driven processes with the intrusion of tropical hypoxic waters into the NBUS and poleward coastal trapped waves as shown in the following section.

#### Relation to remote forcing: Water masses, currents, coastal waves and oxygen supply

The periodical intrusion of tropical water from the Angola Gyre [48, 49] and the interaction of South Atlantic Central Water (SACW) with the East South Atlantic Central Water (ESACW) [67, 68] including the cross-shelf circulation [69] cause oxygen depleted water over the Namibian shelf [70, 71] and anoxic conditions in the bottom water layer [72] supporting the formation of H_2_S enriched bottom water. This water can be the source of sulphur plumes whereas the different processes of H_2_S production were illustrated in the introduction.

The AGCW (Angola Gyre Central Water, sub-type of South Atlantic Central Water) mass fraction in the two depth levels of 63 m and 93 m of Junker et al. [73] is used as proxy for the description of the water masses intrusion coming from the tropical area, exactly from the Angola Gyre (Fig 8A, S6 Table). For the calculation of the AGCW fraction, the authors used in-situ measurements of a mooring (deployed from December 2002 to November 2015) at 23°S and 14°E, located at a water depth of about 130 m and 20 NM offshore of the Walvis Bay. Fig 8A demonstrates the significant correlation between the seasonal climatology of the sulphur plume size and the AGCW mass fraction (63 m: Pearson’s correlation coefficient of 0.84 with a confidence limit of 99.9%; 93 m: Pearson’s correlation coefficient of 0.86 with a confidence limit of 99.9%). A significant correlation is also observed for the climatology of the sulphur plume intensity and the AGCW mass fraction (63 m: correlation coefficient: 0.85, confidence limit: 99.9%; 93 m: correlation coefficient: 0.86, confidence limit: 99.9%). The sulphur plume size increases from January to April with increasing fraction of water mass originating from the Angola Gyre (Fig 8A). The AGCW mass fraction is highest between March (93 m) and April (63 m) at the same time of the maximum of sulphur plume size. For instance, the tropical water accounts for 67% (63 m) or 64% (93 m) of the water mass fraction on the Namibian shelf in April [73]. After this time the sulphur plume size decreases continuously up to December with decreasing AGCW mass fraction at 93 m water depth. The AGCW mass fraction at 63 m water depth decreases much more but only up to September because the intrusion of tropical water mass depends on the water depth [73]. The sulphur plume intensity reaches their maximum already in February, about one (AGCW mass fraction at 93 m) to two months (AGCW mass fraction at 63 m) earlier than the maximum of the fraction of tropical waters originating from the Angola gyre. The intensity of sulphur plumes remains nearly constant between March and June during the decrease of the AGCW mass fraction and is very low between July and October at the same time of the minimum of the AGCW mass fraction. Fig 8A illustrates that the seasonal climatology of the sulphur plume size and intensity is strongly related to the AGCW mass fraction. This correlation can be explained because the oxygen supply on the Namibian shelf is strongly coupled to the fraction of water originating from the Angola Gyre [30]. High percentage values of AGCW mass fraction indicate the intrusion of tropical warm and saline but oxygen depleted water [49] which supports the formation of H_2_S. In the whole coastal area off Namibia, the occurrence of sulphur plumes can be influenced by the intrusion of tropical waters because these waters can be present up to about 27°S [74, 75]. The extend of this intrusion depends on the southward shift of the Angola Benguela Frontal Zone (ABFZ), a frontal feature situated approximately between 15°S and 17°S [50, 76–78]. The southward shift may be associated with an anomalous southward intrusion of warm water which might be depleted in oxygen which supports again the formation of H_2_S [50]. In austral summer, the front reaches its southernmost position, while it is positioned farthest north in winter [79]. It matches very well with the seasonal cycle of sulphur plumes (Fig 5A). The maximum of sulphur plumes is observed at the end of the southernmost intrusion of oxygen depleted water.

The seasonal ventilation of the subsurface water over the Namibian shelf is controlled by local and remote-driven processes like the interplay between the SACW (South Atlantic Central Water) and the ESACW (Eastern SACW) including the cross-shelf circulation [48, 49, 67, 68]. During austral summer SACW is transported southward into the NBUS by the Angola current and the following poleward undercurrent [30]. This hypoxic, nutrient rich water originates from the area of the Angola gyre [80]. During the austral winter the oxygen rich and nutrient poor ESACW is spread northwards by the Benguela current from the area of the Agulhas retroflection zone [81, 82]. From October to April, southward (poleward) directed currents in almost the entire water column are observed in the monthly climatology of the alongshore velocity at the mooring position of 23°S and 14°E [73]. However northward (equatorward) directed currents are observed from May to September. Therefore, the ventilation of the bottom water layer should be worse in the period from October to April than from May to September. The locally forced process of cross-shelf circulation controls the fraction of ESACW on the Namibian shelf [69]. This process can ventilate the shelf water much more efficiently than the advection by the poleward undercurrent [9]. The monthly climatology of the cross-shore velocity at the coastal area of 23°S underlines offshore directed currents in the bottom water layer during January, February and April but onshore directed currents during all other months with an exception in October [73]. It means lower ventilation at the beginning of the year compared to the rest of the year. In the austral summer and autumn at the same time of poleward and offshore directed currents (seen in the monthly climatology of alongshore and cross-shore velocities of Junker et al. [73]), anoxic bottom waters are observed at the central Namibian shelf if the SACW fraction is higher than 55% [30]. We found that the seasonality of SACW fraction including the cross-shore ventilation is very well correlated with the occurrence of coastal sulphur plumes. Indeed, the season of sulphur plumes starts in February and peaks in April during high SACW fractions and low cross-shore ventilation supporting the formation of H_2_S enriched bottom waters.

Coastal trapped waves forced by equatorial Kelvin waves [83, 84] can also play a role for the seasonality of coastal sulphur plumes. They can penetrate the NBUS up to 24°S modulated by local forcing (winds, Benguela current) [83, 84]. These waves present also seasonal and semi-annual cycles as observed in the SLA in Fig 8B (S6 Table) in agreement with other studies [85–87]. In February to March, a downwelling coastal wave arrives with a high SLA following with an upwelling coastal wave in June, July and August and another downwelling coastal wave in September, October and November (Fig 8B). The ABFZ has its southern most position at the same period as the downwelling coastal trapped wave with the penetration of the AGCW (see description above). As shown by Bachèlery et al. [84], a downwelling (upwelling) coastal trapped wave is associated with a decrease (increase) of oxygen in the NBUS due to the slope of the oxycline in the upwelling area associated with advection processes. This downwelling coastal trapped wave might further decrease the oxygen concentration over the continental shelf in February to March and can contribute to the sulphur plume peak event during the austral fall season.

In-situ measurements of dissolved oxygen in the bottom water layer of the Namibian shelf are analyzed to understand the seasonal variability of sulphur plumes. The CARS climatology presents the seasonal cycle of oxygen (Fig 8A) [88]. The oxygen concentrations in the bottom water layer vary seasonally in agreement with previous studies [4, 24] and show phases of hypoxic and anoxic conditions [6, 9, 30, 89] as well as ventilation events [72, 90]. The water column hypoxia reaches its peak in the period of late austral summer to fall with a pronounced oxygen minimum and is weakened in the period of austral winter to early spring [4, 90]. The seasonal variability of oxygen supports the formation of H_2_S in late austral summer to autumn which increases the probability for the occurrence of coastal sulphur plumes during this time period.

### Discussion of annual variability of sulphur plumes

#### Relation to local forcing: Winds and upwelling intensity

**Fig 9:**
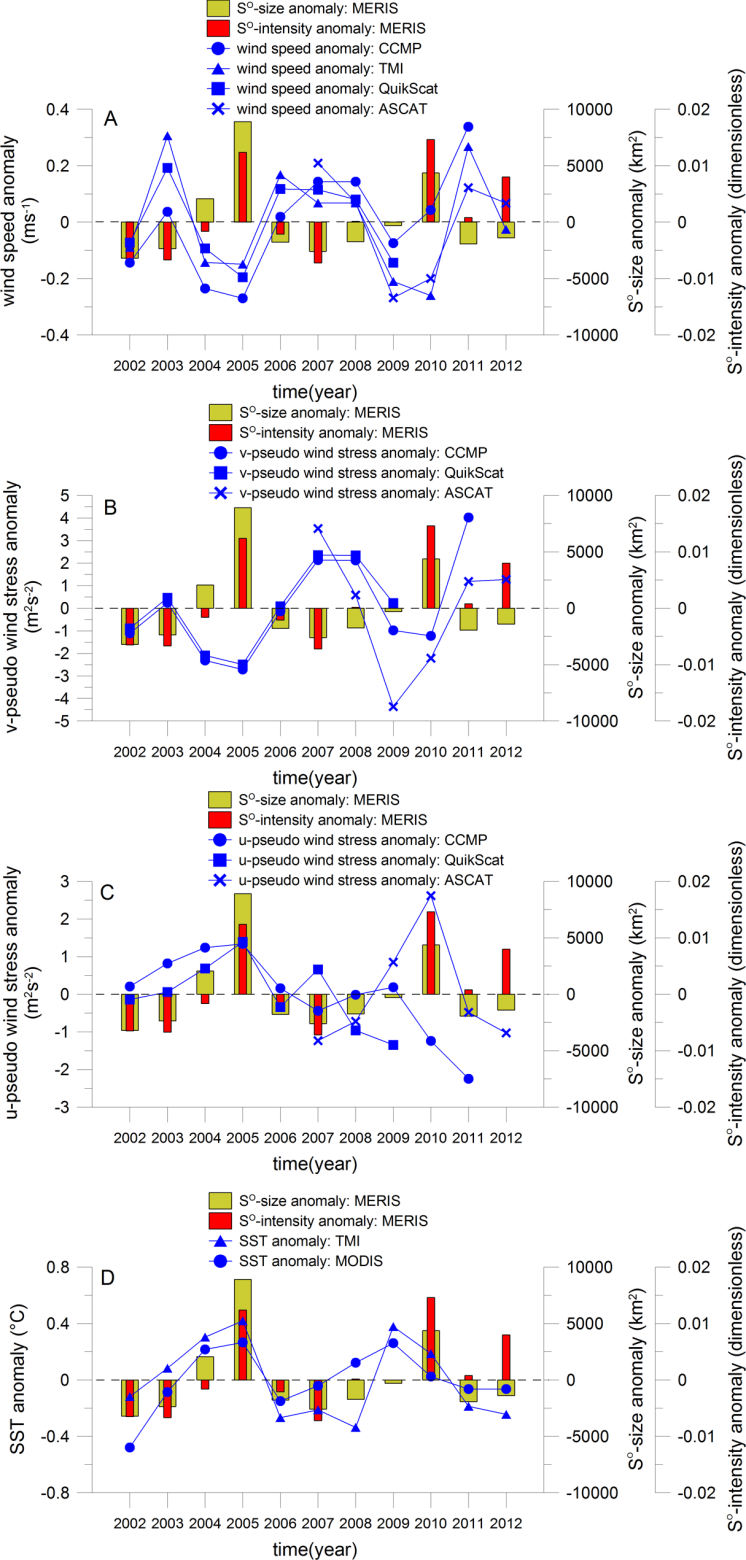
Annual variability of sulphur plumes in relation to local forcing. Annual anomalies of sulphur plume size and intensity, of wind speed at 10 m high of CCMP, TMI, QuikScat and ASCAT (A), of pseudo wind stress v-component at 10 m high of CCMP, QuikScat and ASCAT (B), of corresponding pseudo wind stress u-component (C) and of sea surface temperature of TMI and MODIS (D). The annual anomalies were calculated from the difference of yearly means and the global mean of years 2002 to 2012.

The analysis of the sulphur plumes in relation to the annual wind conditions is based on Fig 9A–9C (S7 Table). Positive anomalies of the sulphur plume size (years 2004, 2005 and 2010) are observed during negative anomalies of wind speed and northward alongshore pseudo wind stress as well as during positive anomalies of westwards cross shore wind component in the NBUS. Indeed, for the years with a decreased upwelling, the ventilation of the bottom water by mixing and by the onshore Ekman recirculation is decreased as well as the discharge of the H_2_S enriched bottom water pool is probably decelerated. This leads to an enhanced time period of H_2_S formation in the bottom water and an increased size of sulphur plumes. Lamont et al. [91] pointed out also the years 2005 and 2010 with the lowest number of upwelling days and the largest number of downwelling days per year over the 1979–2015 period. Negative anomalies of the sulphur plume size (years 2003, 2006 to 2008, 2011, 2012) are mainly observed during positive anomalies of wind speed and northward alongshore pseudo wind stress indicating to intensified upwelling conditions. This probably increases the ventilation of the bottom water by mixing and onshore Ekman recirculation and also accelerates the discharge of the pool of H_2_S enriched bottom water leading to a consecutive reduced time period for the occurrence of sulphur events as well as to a decreased yearly size of sulphur plumes. Exceptions are the years 2002 and 2009 where remote-driven processes could play the main role. The local wind conditions can also influence the annual variability of the sulphur plume intensity (Fig 9A–9C). There is an enhanced probably for high and low intensities of sulphur plumes during years with decreased and enhanced wind speed as well as northward alongshore pseudo wind stress, respectively.

The satellite SST measurements are used as proxy for the annual variability of upwelling intensity as explained before, and thus to investigate the relations to sulphur plumes (Fig 9D, S7 Table). The annual anomalies of upwelling intensities derived from SST products of MODIS (blue curve with dots in Fig 9D) and TMI (blue curve with triangles) correlate well (Pearson’s correlation coefficient of 0.60 with a confidence limit of 99.7%). Positive anomalies of the sulphur plume size are observed for the years 2004, 2005 and 2010 at the same time of positive SST anomalies in the NBUS meaning in years with decreased upwelling intensity. Negative anomalies of the sulphur plume size are observed in the years 2002, 2003, 2006 to 2008 as well as 2011 and 2012 at the same time of negative SST anomalies in the NBUS meaning in years with enhanced upwelling (with the exception of year 2009). Both findings verify the strong influence of local wind driven upwelling process on the activity of sulphur plumes. The size of sulphur plumes is higher than normal for the years with reduced upwelling intensity. This lead probably to a decreased lateral ventilation of bottom water layer providing anoxic conditions and the formation of H_2_S [92] as well as to a deceleration of the discharge of the H_2_S enriched bottom water pool. The upwelling intensity influences also the annual variability of the sulphur plume intensity (Fig 9D). This is particularly apparent for the years where the annual anomalies of sulphur plume intensity correlate to the corresponding anomalies of their size. For example, the anomalies of sulphur plume intensity are positive (e.g. years 2005 and 2010) and negative (e.g. years 2002, 2003, 2006 and 2007) for the same years like the anomalies of sulphur plume size as well as at the same time of decreased and enhanced upwelling, respectively. However there are exceptions to this rule for the years 2004, 2008, 2011 and 2012. For these years their intensity is not only influenced by the physical process of upwelling but probably by biogeochemical processes supporting the transformation of hydrogen sulphide to sulphur in the water column.

In summary, the local-driven wind processes have an influence on the annual variability of sulphur plumes. The sizes and intensities of sulphur plumes can be high and low during years with decreased (decreased mixing and lateral ventilation providing anoxic conditions) and enhanced upwelling intensity (increased mixing and lateral ventilation providing well-ventilated bottom water), respectively.

#### Relation to remote forcing: Water masses, currents, coastal waves and oxygen supply

The discussion about the relations between the annual variability of the SACW fraction and the sulphur plumes is based on different studies [30, 69, 89, 93]. The authors of these papers observed a well pronounced annual variability of the SACW fraction for the period from 2002 to 2012. The comparison with the annual variability of sulphur plumes shows that the years with higher and lower SACW fractions in the bottom water layer over the Namibian shelf correlate to higher and lower sizes of sulphur plumes, respectively. The strongest sulphur plume season of year 2005 (Fig 6A) is associated with the highest SACW fraction of the studied period. In March to April of this year, a maximum SACW fraction of 85% is reached [89]. It is much more than the threshold value of 55% assumed to be a precondition for the development of anoxic bottom waters [30]. In the year 2004, the maximum SACW fraction is also high during the sulphur plume season. About 70% is observed [30]. It corresponds to the third highest observed size of sulphur plumes in 2004. In contrast, a maximum SACW fraction of only 60% is reached in March to April 2008 as well as in February 2011 [89, 93]. This is rather low compared to years of 2004 and 2005. The maximum SACW fractions of 2008 and 2011 are only 5% above the mentioned limit of 55% which could explain the weaker activity of sulphur plumes in these years.

**Fig 10:**
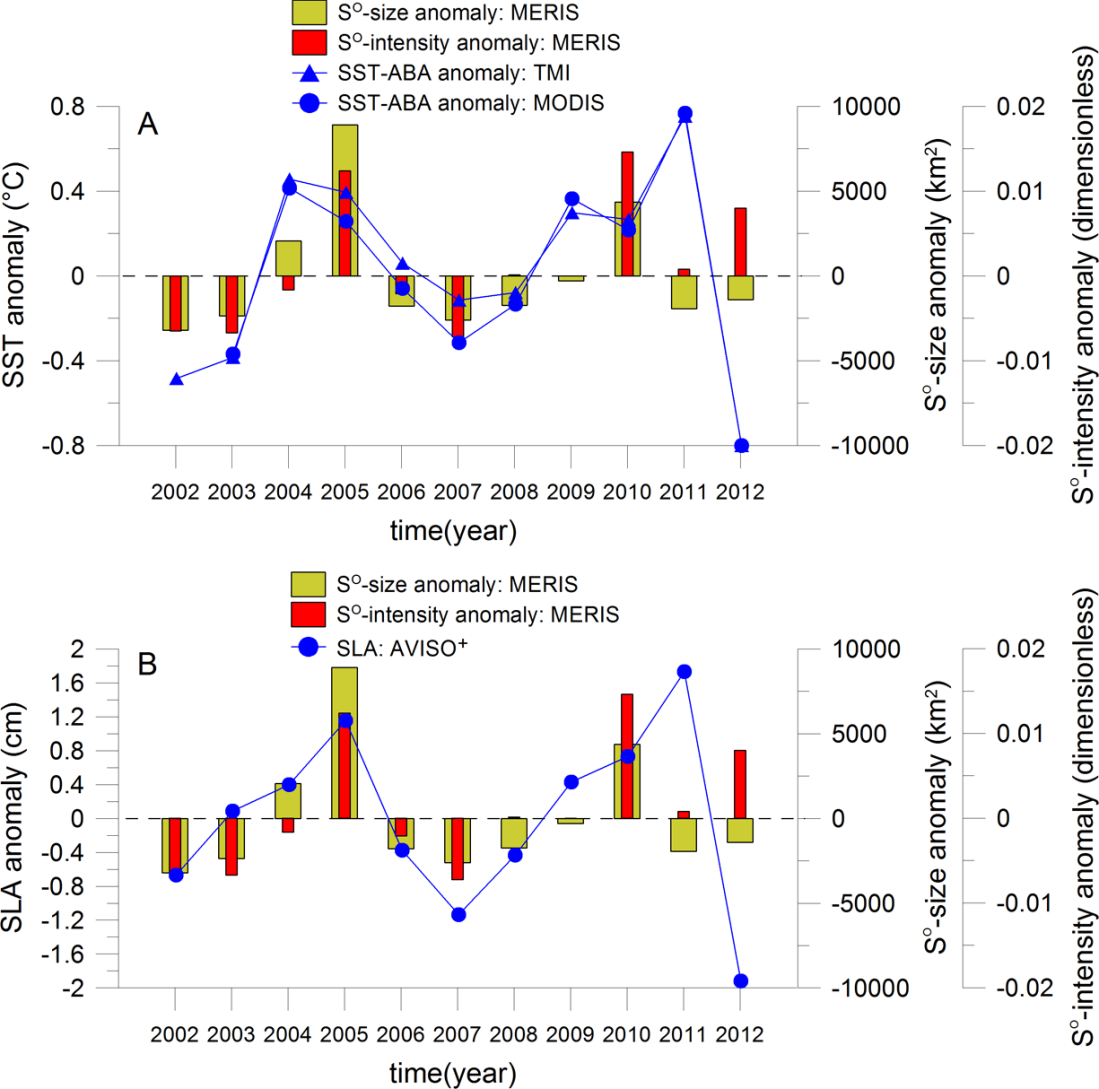
Annual variability of sulphur plumes in relation to remote forcing. Annual anomalies of sulphur plume size and intensity, of TMI and MODIS SST over the Angola-Benguela area (A) and of sea level anomaly between 18°S and 19°S within the 1° width coastal band (B). The SLA positive trend from 2002 to 2012 was removed.

Annual ABA-SST anomalies of MODIS and TMI in the ABA (Angola Benguela Area, 19.5°S-10.5°S and 8.5°E-15.5°E) (Fig 10A, S8 Table) are used as proxies for the intrusion of oxygen depleted water into the Namibian shelf area. A significant time lag of about one to two months between the ABA-SST index of Florenchie et al. [94, 95] (leading signal) and the monthly annual anomalies of sulphur plume size is observed. This indicates that the activity of sulphur plumes depends among others from remotely driven processes coming from the ABA area with a time delay between one to two months. It corresponds also to the fact that the water masses from the ABA reach the Walvis Bay region in about one to two months if a mean current velocity between 17 cm s^-1^ and 34 cm s^-1^ is assumed. Such current velocities are in the range of measurements and modeling estimations [80, 96–99]. Therefore, only the two months period (November to December) before the start of the sulphur plume season (February to April) is included in the calculation of the annual ABA-SST anomalies (Fig 10A). The annual ABA-SST anomalies of MODIS (blue curve with dots in Fig 10A) and TMI (blue curve with triangles) correlate with a Pearson’s correlation coefficient of 0.79 and a 99.9% confidence limit. Positive annual anomalies of the sulphur plume size are observed (years 2004, 2005 and 2010) during positive ABA-SST anomalies meaning during environmental conditions with enhanced intrusion of tropical warm and saline but oxygen depleted water which supports the production of H_2_S. It is confirmed with the oxygen concentrations in the deep water off Walvis Bay which were lower in austral summer to early spring of the year 2005 compared to the years between 2002 and 2006 [4] and the years between 2002 and 2012 [100] (with the exception for the year 2007). Negative annual anomalies of the sulphur plume size are derived (years 2002, 2003, 2006 to 2008 and 2012) during negative SST anomalies in the ABA meaning in years with reduced intrusion of tropical water. The reduced sulphur plume size in the year 2002 can be explained by this remote forcing which increases the oxygen level and reduces the production of H_2_S. Exceptions are the years 2009 and 2011 where such correlations between the ABA-SST anomalies and the anomalies of sulphur plume size are not found (Fig 10A). This indicates that local driven processes should have more influence than the remote driven process. This is evident for the year of 2011 where the enhanced upwelling intensity (cf. Fig 9) causes increased ventilation in the bottom water layer which reduces the probability of the formation of H_2_S.

The annual variability of the sulphur plume intensity is also influenced by the remote driven process (Fig 10A). This is evident for years where the annual anomalies of sulphur plume intensities correlate to the corresponding anomalies of their size. For example, the anomalies of sulphur plume intensities are positive (e.g. years 2005 and 2010) and negative (e.g. years 2002, 2003, 2006 and 2007) in the same years like the anomalies of sulphur plume size meaning at the same time of enhanced and decreased intrusion of tropical water, respectively. However there are exceptions to this rule for the years like 2004, 2008, 2011 and 2012 where probably more local processes influence the sulphur plume intensity.

Coastal trapped waves can also influence annual variability of sulphur plumes. At special conditions the signatures of coastal trapped waves on currents are observed as far as 30°S [83]. As explained for the seasonal cycle, a downwelling wave has a negative impact on oxygen in the NBUS due to the interaction of the vertical and horizontal advection with the slope of the oxycline in the upwelling area [83]. The annual variability of the SLA is presented in Fig 10B (S8 Table). Only the three months period (November to January) was included in the calculation of the annual SLA anomalies. This period describes the arrival of coastal trapped waves into the study area and is before the start of the sulphur plume season (February to April). The highest positive anomalies of the sulphur plume size are found during the highest SLA values (2004, 2005 and 2010). During these years, the downwelling coastal trapped waves are stronger and can decrease the oxygen concentration in the NBUS. These conditions can favour the appearance of sulphur plumes. Negative annual anomalies of the sulphur plume size are observed (years 2002, 2006 to 2008 and 2012) during negative SLA values meaning in years with enhanced upwelling coastal trapped waves. This increases the oxygen concentration on the Namibian shelf and might further decrease the sulphur plume size. Exceptions are the years 2009 and 2011 where such relations between the SLA values and the anomalies of sulphur plume size are not found. These exceptions were already observed in the ABA-SST anomalies (cf. Fig 10A and 10B).

Depending of the years, the relative contribution of the local wind-driven forcing and the remote equatorial forcing varies and can explain the annual sulphur plume variability for the studied MERIS period, except the year 2009 for which other processes might be dominant (e.g. coupled physical/biogeochemical processes in the water column and/or in the sediment layers not seen with satellite sensors).

## Summary and conclusions

For the first time we have proved the seasonal variability of coastal surface sulphur plumes with the long-term satellite data set of MERIS. In the past different studies described their seasonality based on short time periods. However, the main and off-seasons were not well specified. We found that the sulphur plumes have a strong seasonal cycle with pronounced main and off-seasons. The main season (e.g. high sulphur plume activity) is in late austral summer and early austral autumn (February up to May). The sulphur plume size continuously decreases up to the off-season in austral spring and early austral summer (September up to December). The seasonal cycle of sulphur plume intensity is only slightly different. The highest and lowest median intensities are observed in February and in October, respectively. For the first time the seasonal cycles of sulphur plume size and intensity are also explained in relation to local and remote-driven processes. Our study illustrates that the main season is at the same time of increasing equatorward alongshore winds, so before the main upwelling season. Consequently, the coastal upwelling and offshore Ekman transport are increased which enhances the probability of the upwelling of H_2_S enriched bottom water. Furthermore, the activity of sulphur plumes is high during the seasonal oxygen minimum time period supporting the accumulation of H_2_S in the bottom water layer. The oxygen level in the bottom water is controlled by different local and remote-driven processes. Therefore, the main season of sulphur plumes is observed at the same time of the seasonal reduction of cross-shore ventilation of the bottom waters, the seasonal southernmost position of the ABFZ, the seasonal maximum of mass fractions of SACW and AGCW (sub-type of SACW) as well as the seasonal arrival of the downwelling coastal trapped waves. In contrast, the off-season is found during the upwelling season with maximum upwelling intensity and enhanced oxygen supply on the Namibian shelf. This higher oxygen level is associated with an increased ventilation of the bottom water by local processes (Ekman recirculation, mixing) as well as remote driven processes with the equatorward position of the ABFZ, the seasonal minimum of SACW mass fraction and upwelling coastal trapped waves.

The annual variability of coastal surface plumes was never investigated before, especially not with the long-term satellite data of MERIS. We conclude that the annual variability of sulphur plumes is characterized by years with very high activity interrupted by periods of lower activities. The largest sizes of sulphur plumes over the 2002–2012 time period are observed in the years 2004, 2005 and 2010. Lower sizes are found in the intermediate time periods: 2002 to 2003, 2006 to 2009 and 2011 to 2012. The highest and lowest annual median intensities are observed in the years 2010 and 2007, respectively. For the first time this study highlights that the annual variability can be explained by the superposition of local and remote-driven processes. The sulphur plume activity is high in years with decreased yearly mean of the equatorward alongshore winds and increased coastal SST compared to normal years. It means years with a lower yearly mean of upwelling intensity probably supporting the deceleration of the depletion of the yearly H_2_S pool. There is a higher probability for an enhanced sulphur plume activity in years with a decreased oxygen supply promoting the formation of H_2_S. The years with higher sulphur plume activity are associated with decreased lateral ventilation of bottom waters by local processes as mixing and Ekman recirculation. Furthermore, the remote-driven processes like the displacement of the ABFZ, the interaction of SACW with the ESACW and the coastal trapped waves play also a strong role for the oxygen water content on the Namibian Benguela shelf. Therefore, the probability of the occurrence of strong sulphur plume activity is enhanced in years with a more southern position of the ABFZ, an increased mass fraction of SACW and stronger downwelling coastal trapped waves.

Overall, our study illustrates the dominant role of local forcing as well as remote forcing on the seasonal and annual variability of sulphur plumes. The complex interplay between these two types of processes provides their variability. Our study might help in the future to monitor and manage these toxic events and last but not least to forecast them.

## Supporting information

S1 Document Investigation of the cloud impact on the seasonal and annual detection of sulphur patches in the study area. (PDF)

S2 Document Spectral properties of sulphur plumes compared to different Namibian Benguela optical water types. (PDF)

S1 Table Sulphur spectra measured by different satellite sensors: MODIS, MERIS, HICO and MSI. (XLS)

S2 Table Sulphur plume intensity of all identified sulphur pixels for the 2002–2012 time period. Statistics of sulphur plume intensity. (XLS)

S3 Table Seasonal variability of sulphur plume size and intensity. (XLS)

S4 Table Annual variability of sulphur plume size and intensity. (XLS)

S5 Table Seasonal variability of sulphur plumes in relation to local forcing. (XLS)

S6 Table Seasonal variability of sulphur plumes in relation to remote forcing. (XLS)

S7 Table Annual variability of sulphur plumes in relation to local forcing. (XLS)

S8 Table Annual variability of sulphur plumes in relation to remote forcing. (XLS)
