# Supplementary material for: Seasonal and annual variability of coastal sulphur plumes in the northern Benguela upwelling system
Source: PLoS One. 2018 Feb 8;13(2):e0192140. doi: 10.1371/journal.pone.0192140 (PMC5805238; doi:10.1371/journal.pone.0192140)
Supplement: S1 Document — (PDF) [file pone.0192140.s001.pdf]

Table 1. Investigation of the cloud impact on the seasonal and annual detection of sulphur patches in the study area. The first part of the table represents the seasonal impact. For each month of the monthly climatology, the number of days per month (Ndm in days), the mean area-averaged cloud fraction (Mcfm) and their standard deviation (σ_{cfm}), the number of days where the cloud fraction is above 2/3 on 3 consecutive days (Ncfm3d in days) and the corresponding percentages in relation to the number of days in the month (Ncfm3d in %) are given. The second part of the table represents the annual impact. For each year, the number of days per year (Ndy in days), the mean area-averaged cloud fraction (Mcfy) and their standard deviation (σ_{cfy}), the number of days where the cloud fraction is above 2/3 on 3 consecutive days (Ncfy3d in days) and the corresponding percentages in relation to the number of days in the year (Ncfy3d in %) are given.

month	Ndm in days	Mcfm	σ_{Cfm}	Ncfm3d in days	Ncfm3d in %
Jan	339	0.65	0.22	82	24.2
Feb	311	0.66	0.21	78	25.1
Mar	333	0.57	0.25	39	11.7
Apr	329	0.47	0.28	30	9.1
May	341	0.43	0.25	11	3.2
Jun	330	0.40	0.28	7	2.1
Jul	341	0.42	0.30	13	3.8
Aug	342	0.54	0.30	33	9.6
Sep	330	0.59	0.28	66	20.0
Oct	341	0.57	0.27	40	11.7
Nov	331	0.54	0.26	29	8.8
Dec	334	0.59	0.23	46	13.8
range	311-342	0.40-0.66	0.21-0.30	7-82	2.1-25.1
year	Ndy in days	Mcfy	σ_{Cfy}	Ncfy3d in days	Ncfy3d in %
2002	365	0.52	0.27	43	11.8
2003	365	0.51	0.27	34	9.3
2004	366	0.54	0.28	44	12.0
2005	365	0.54	0.25	41	11.2
2006	365	0.53	0.29	59	16.2
2007	365	0.54	0.27	37	10.1
2008	366	0.54	0.27	49	13.4
2009	365	0.53	0.27	37	10.1
2010	365	0.55	0.29	59	16.2
2011	365	0.54	0.28	53	14.5
2012	366	0.53	0.27	25	6.8
range	365-366	0.51-0.55	0.25-0.29	25-59	6.8-16.2
