# Supplementary material for: Seasonal and annual variability of coastal sulphur plumes in the northern Benguela upwelling system
Source: PLoS One. 2018 Feb 8;13(2):e0192140. doi: 10.1371/journal.pone.0192140 (PMC5805238; doi:10.1371/journal.pone.0192140)
Supplement: S2 Document — (PDF) [file pone.0192140.s002.pdf]

The method for the identification of sulphur plumes is based on the different optical water properties [1]. The sulphur plumes in the Namibian coastal area are clearly distinguished from other optical water masses by their special spectral signatures (Fig 1, Tab 1). The spectral peaks of unaffected onshore and offshore waters, algae blooms and river plumes vary between the blue and green wavelength range depending on the composition of optically active water constituents like chlorophyll-a, yellow substances and suspended matter. In contrast to the sulphur plumes, most of these unaffected spectra are influenced by the absorption of chlorophyll-a at MERIS bands of 442.4 and 664.6 nm. The offshore waters with low chlorophyll-a concentration are identified by their maxima in the blue MERIS channel of 412.5 nm. Water masses with high concentrations of suspended or resuspended matter like the river plume of the Orange River and the nearest coastal located onshore waters are differentiated from sulphur plumes by their different spectral slopes in the red wavelength range.

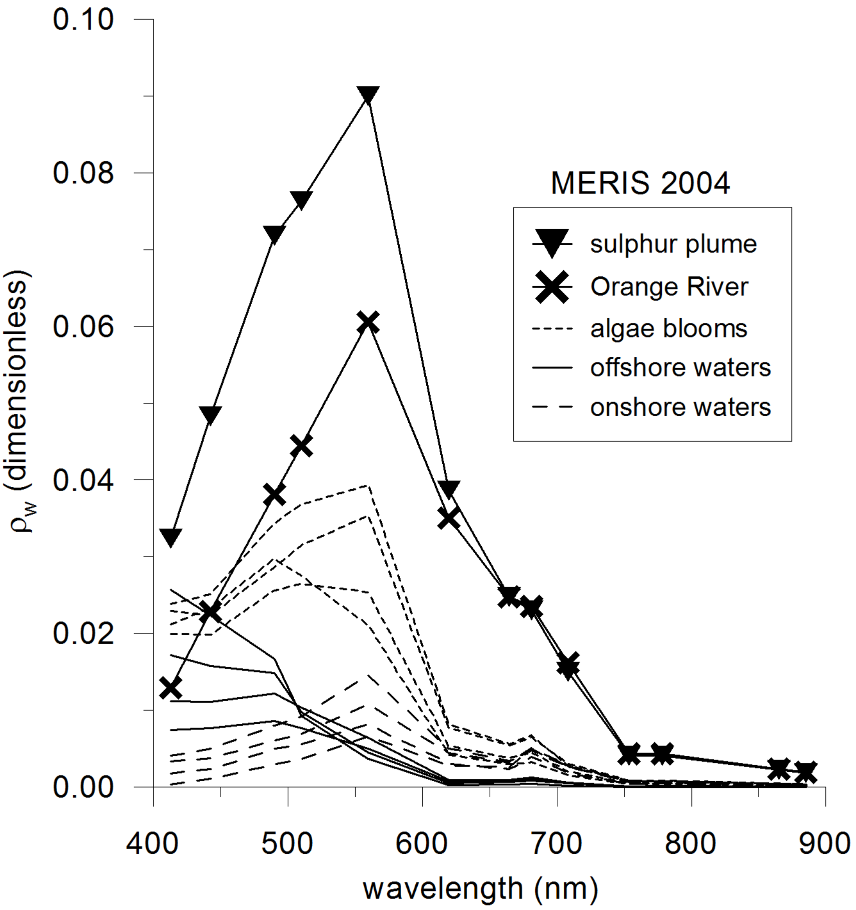

Fig 1. Comparison of spectral properties of different optical water masses. The mean spectral curves represent the optical water masses like onshore and offshore waters, algae blooms, river discharges and sulphur plumes in 2004.

Table 1. Spectral data.

MERIS wavelength/nm	sulphur plume reflectance/dimensionless	Orange river reflectance/dimensionless	1-algal bloom reflectance/dimensionless	2-algal bloom reflectance/dimensionless	3-algal bloom reflectance/dimensionless
413	0.03234	0.01293	0.02382	0.02296	0.01993
442	0.04830	0.02283	0.02513	0.02238	0.01984
490	0.07177	0.03816	0.03435	0.02864	0.02561
510	0.07630	0.04442	0.03680	0.03146	0.02648
560	0.08995	0.06052	0.03934	0.03541	0.02540
620	0.03865	0.03503	0.00818	0.00772	0.00535
665	0.02477	0.02480	0.00564	0.00542	0.00379
681	0.02303	0.02351	0.00673	0.00649	0.00451
708	0.01505	0.01622	0.00281	0.00305	0.00203
753	0.00409	0.00433	0.00070	0.00085	0.00052
778	0.00413	0.00432	0.00079	0.00086	0.00058
865	0.00225	0.00234	0.00043	0.00047	0.00032
885	0.00183	0.00191	0.00035	0.00039	0.00026

MERIS wavelength/nm	4-algal bloom reflectance/dimensionless	1-offshore water reflectance/dimensionless	2-offshore water reflectance/dimensionless	3-offshore water reflectance/dimensionless	4-offshore water reflectance/dimensionless
413	0.02117	0.02569	0.01714	0.01121	0.00744
442	0.02334	0.02236	0.01575	0.01107	0.00767
490	0.02973	0.01670	0.01484	0.01219	0.00858
510	0.02753	0.00927	0.00976	0.01034	0.00765
560	0.02108	0.00366	0.00454	0.00639	0.00502
620	0.00421	0.00024	0.00052	0.00096	0.00080
665	0.00299	0.00033	0.00067	0.00093	0.00071
681	0.00322	0.00040	0.00083	0.00129	0.00102
708	0.00153	0.00016	0.00047	0.00062	0.00061
753	0.00039	0.00005	0.00011	0.00006	0.00008
778	0.00043	0.00004	0.00013	0.00016	0.00016
865	0.00024	0.00002	0.00007	0.00009	0.00009
885	0.00019	0.00002	0.00006	0.00007	0.00007

MERIS wavelength/nm	1-onshore water reflectance/dimensionless	2-onshore water reflectance/dimensionless	3-onshore water reflectance/dimensionless	4-onshore water reflectance/dimensionless
413	0.00410	0.00337	0.00179	0.00032
442	0.00498	0.00377	0.00232	0.00107
490	0.00802	0.00608	0.00497	0.00298
510	0.00920	0.00693	0.00562	0.00363
560	0.01456	0.01076	0.00818	0.00658
620	0.00502	0.00443	0.00305	0.00280
665	0.00339	0.00320	0.00234	0.00261
681	0.00472	0.00510	0.00393	0.00499
708	0.00258	0.00268	0.00183	0.00264
753	0.00069	0.00084	0.00040	0.00077
778	0.00072	0.00076	0.00052	0.00073
865	0.00040	0.00042	0.00029	0.00040
885	0.00033	0.00035	0.00023	0.00033

References

1. Ohde T, Siegel H, Reißmann J, Gerth M. Identification and investigation of sulphur plumes along the Namibian coast using the MERIS sensor. Continental Shelf Research. 2007; 27:744-756.
